# Evaluation of drought-tolerant varieties based on root system architecture in cotton (*Gossypium hirsutum* L.)

**DOI:** 10.1186/s12870-024-04799-x

**Published:** 2024-02-21

**Authors:** Congcong Guo, Lingxiao Zhu, Hongchun Sun, Qiucheng Han, Shijie Wang, Jijie Zhu, Yongjiang Zhang, Ke Zhang, Zhiying Bai, Anchang Li, Liantao Liu, Cundong Li

**Affiliations:** 1 State Key Laboratory of North China Crop Improvement and Regulation/Key Laboratory of Crop Growth Regulation of Hebei Province/College of Agronomy, Hebei Agricultural University, Baoding, Hebei 071001 China; 2 Handan Academy of Agricultural Sciences, Handan, 056001 China; 3 Institute of Cereal and Oil Crops, Hebei Academy of Agriculture and Forestry Sciences, Shijiazhuang, 050051 China

**Keywords:** Drought, Cotton, Principal component analysis, Root traits, Drought tolerance index

## Abstract

**Background:**

Root system architecture (RSA) exhibits significant genetic variability and is closely associated with drought tolerance. However, the evaluation of drought-tolerant cotton cultivars based on RSA in the field conditions is still underexplored.

**Results:**

So, this study conducted a comprehensive analysis of drought tolerance based on physiological and morphological traits (i.e., aboveground and RSA, and yield) within a rain-out shelter, with two water treatments: well-watered (75 ± 5% soil relative water content) and drought stress (50 ± 5% soil relative water content). The results showed that principal component analysis identified six principal components, including highlighting the importance of root traits and canopy parameters in influencing drought tolerance. Moreover, the systematic cluster analysis was used to classify 80 cultivars into 5 categories, including drought-tolerant cultivars, relatively drought-tolerant cultivars, intermediate cultivars, relatively drought-sensitive cultivars, and drought-sensitive cultivars. Further validation of the drought tolerance index showed that the yield drought tolerance index and biomass drought tolerance index of the drought-tolerant cultivars were 8.97 and 5.05 times higher than those of the drought-sensitive cultivars, respectively.

**Conclusions:**

The RSA of drought-tolerant cultivars was characterised by a significant increase in average length-all lateral roots, a significant decrease in average lateral root emergence angle and a moderate root/shoot ratio. In contrast, the drought-sensitive cultivars showed a significant decrease in average length-all lateral roots and a significant increase in both average lateral root emergence angle and root/shoot ratio. It is therefore more comprehensive and accurate to assess field crop drought tolerance by considering root performance.

**Supplementary Information:**

The online version contains supplementary material available at 10.1186/s12870-024-04799-x.

## Background

The global greenhouse effect has increased the risk of short-term extreme weather events in agriculture, with drought emerging as a primary constraint on crop productivity [1, 2]. Repeated droughts have resulted in a remarkable 50% decline in the average productivity of major global crops [3, 4]. In addition, the world population is expected to reach 10 billion by 2050 [5], resulting in a doubling of global crop production. Cotton (*Gossypium hirsutum* L.) is a pivotal economic crop that exhibits high sensitivity to abiotic stress [6]. Consequently, drought stress has emerged as the predominant stressor during the cotton growing season. Addressing these challenges, developing drought tolerance cotton cultivars adapted to frequent drought stress conditions and identifying indicators of drought tolerance in cotton represent indispensable strategies of paramount strategic significance for the future of agricultural development.

The study of drought tolerance in cotton has been an important topic in the field. Several studies have made progress in this area. For instance, Zou et al. [7] identified parameters such as Fv/Fm, stem water content, leaf water potential, leaf proline content, and leaf malondialdehyde as viable metrics for assessing drought tolerance. Similarly, Quevedo et al. [8] determined that leaf relative water content, net photosynthesis, stomatal conductance, electron transport rate, photochemical quenching, and PSII photochemical efficiency serve as indicators of drought tolerance in cotton. In particular, previous research has mainly focused on using indicators of above-ground parts or conducted indoor studies, often overlooking the significance of the root system. However, it's crucial to note that the root system, being the primary organ for water and nutrient uptake, plays a key role in the drought tolerance of crops.

Roots, as the primary organs for detecting drought signals, play a pivotal role in plant anchoring and in the uptake, storage, and transport of water and nutrients [9, 10]. Root system architecture (RSA) plays a critical role in soil resource acquisition, plant growth, and crop performance [11, 12] and has been hailed as the second green revolution in crop improvement [13]. RSA exhibit extensive phenotypic and genetic diversity [14, 15]. During drought stress, accurately delineating RSA's growth structure and spatial distribution in the soil can enhance the efficient extraction of water and nutrients from resource-limited soil, subsequently facilitating their distribution to the upper parts of the plant through signal transduction [16–20]. Consequently, well-developed root systems in cultivars result in higher yields under drought stress [21].

There are currently many cultivars of cotton in production, each with different levels of stress tolerance. Moreover, these cultivars employ different standards or methods to evaluate their stress tolerance levels. Presently, there is a widely held belief in the utility of employing a multifaceted approach encompassing various multivariate statistical methods, including correlation analysis, membership function analysis, factor analysis, principal component analysis (PCA), grey relational analysis, membership function [22], cluster analysis, and stepwise regression analysis [23]. Among these, the technique utilizing the drought tolerance comprehensive evaluation values (D-value) as the pivotal indicator for assessing drought tolerance is considered more dependable [24]. This preference arises from its incorporation of the interrelationships between various indicators and the consideration of their respective significance. Consequently, the D-value has been frequently adopted by previous researchers for evaluating crop drought tolerance [25, 26].

In summary, previous studies had limitations in comprehensively assessing drought tolerance in cotton, neglecting the significance of root traits in crop drought tolerance. The expansion of root-related phenotypic traits aims to enhance the selection process for drought tolerant cultivars, making it more rigorous and reliable. Recognizing this gap, this study aims to rectify past limitations by integrating a wide array of root indicators into the drought-tolerance cultivars screening process in cotton. Based on field experiments and introducing a wider range of root system indicators, it will provide a new perspective and a more reliable method for screening drought-tolerance cotton cultivars. Thus, this study pursues three specific objectives: (1) to employing multivariate statistical approaches such as PCA, membership function analysis, and cluster analysis to assess cotton's drought tolerant performance under field conditions; (2) to validate the evaluation results using the drought tolerance index of yield and biomass; and (3) to explore the significance of root traits in identifying drought-tolerant cultivars. These findings provide a robust foundation for refining breeding strategies and agricultural practices, aiming to develop cotton cultivars resilient to fluctuating climatic conditions, thereby enhancing sustainable cotton production.

## Results

### Representative image and trait analysis of cotton

**Fig. 1:**
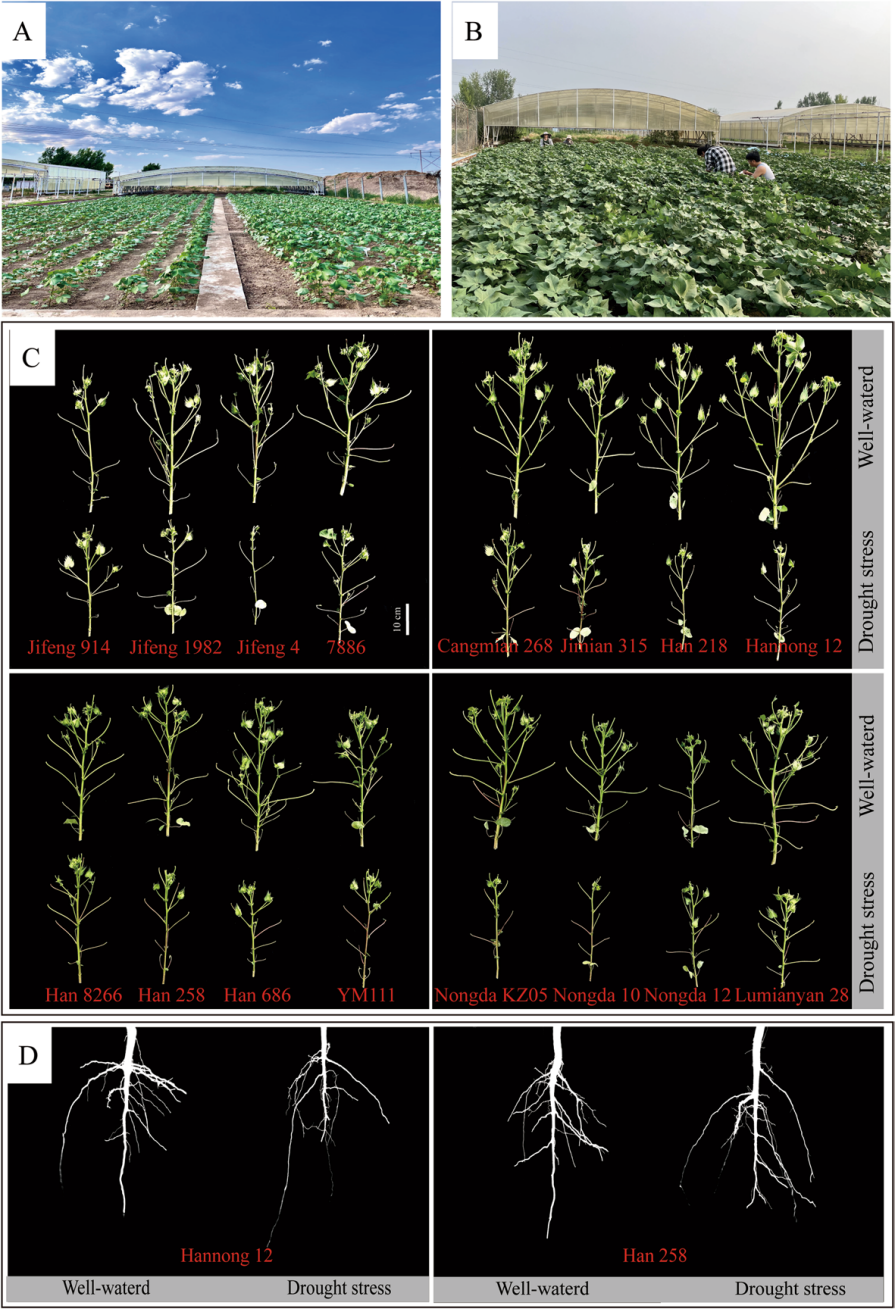
The growth of different cotton cultivars. The growth of the cotton seedling stage (**A**) and the measurement of indicators are carried out in the field (**B**). Representative images of above-ground (**C**) and root (**D**) traits in cotton

Drought stress had significant effects on above-ground and root traits in cotton (Fig. 1). As indicated in Supplementary Tables 1 and 2, among the above-ground traits, leaf area, plant height, SPAD, leaf water potential, and relative water content displayed diminishing tendencies in response to drought stress. In contrast, canopy temperature exhibited an increasing pattern. In terms of root traits, root dry weight, root surface area, root volume, average lateral root emergence angle, average lateral root tip angle, and lateral root count demonstrated declining trends in response to drought stress. Meanwhile, average length—all roots, average length—lateral roots, width/depth ratio, specific root length, and specific root surface area displayed diminishing tendencies. The coefficients of variation for the measured traits ranged from 1.47 to 57.40 in 2021 and from 0.70 to 60.44 in 2022, with most traits exceeding 20% in both years (Supplementary Tables 1 and 2).

**Fig. 2:**
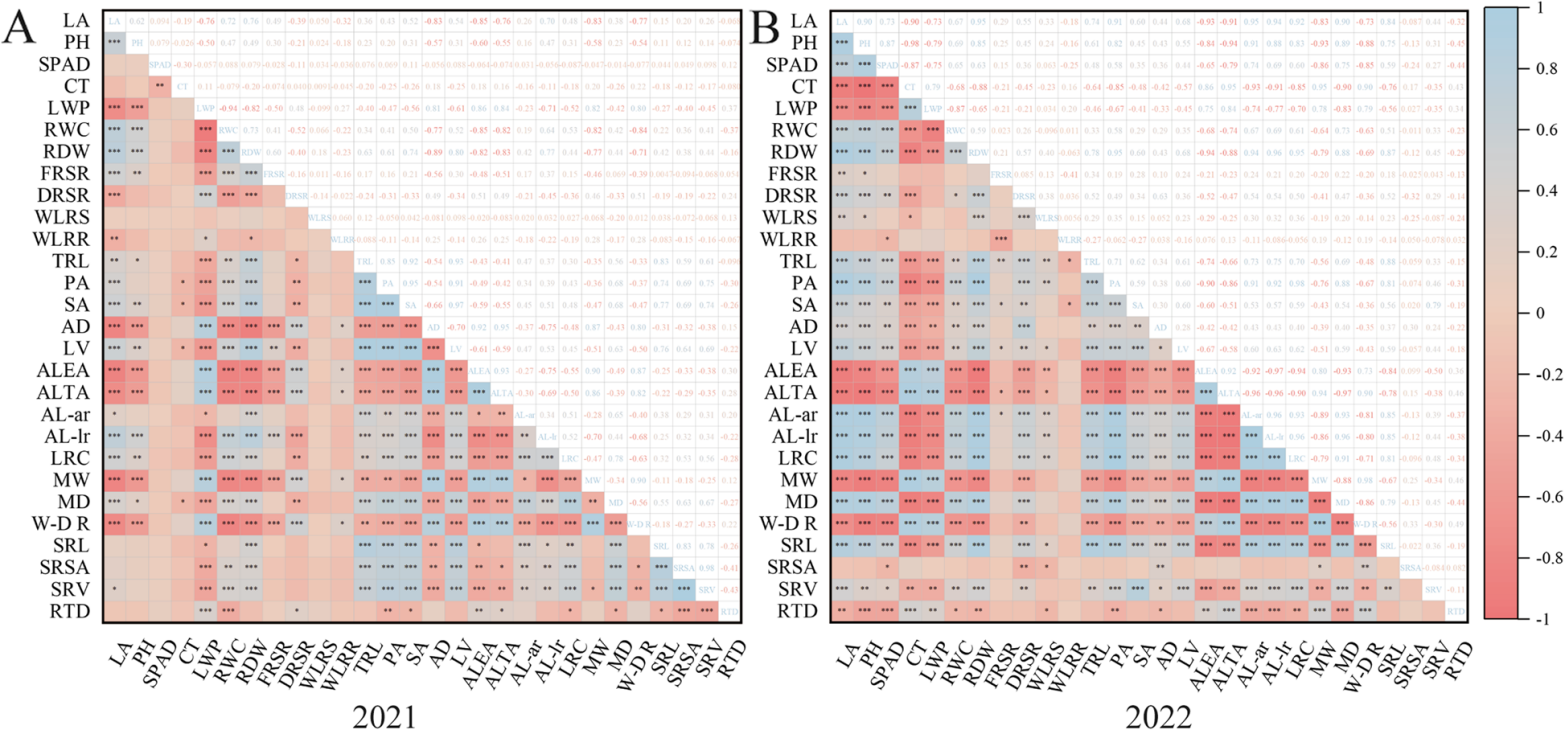
Correlation analysis between the coefficient of tolerance to drought of each trait of cotton in the years 2021 (**A**) and 2022 (**B**). LA, leaf area; PH, plant height; CT, canopy temperature; LWP, leaf water potential; RWC, relative water content; RDW, root dry weight; FRSR, fresh root/shoot ratio; DRSR, dry root/shoot ratio; WLRS, water loss rate of shoot; WLRR, water loss rate of root; TRL, total root length; PA, projection area; SA, surface area; AD, average diameter; AV, average volume; ALEA, average lateral root emergence angle; ALTA, average lateral root tip angle; AL-ar, average length—all roots; AL-lr, average length—all lateral roots; LRC, lateral root count; MW, maximum width; MD, maximum depth; W/D R, width/depth ratio; SRL, specific root length; SRSA, specific root surface area; SRV, specific root volume; RTD, root tissue density. *, ** and ***, significant at 95, 99% and 99.9% confidence levels, respectively. ns, not significant

Spearman's correlation analysis, which revealed strong correlations between different traits, resulted in data redundancy and potentially undermined an accurate assessment of drought tolerance in cotton (Fig. 2). Consequently, we conducted comprehensive evaluations using PCA and cluster analysis.

### PCA of each traits

Six principal components were extracted for both growing seasons (Tables 1 and 2). In 2021, the contribution rates of the top six comprehensive evaluation indicators, Cl Composite Index (Cl1-Cl6), were 45.70%, 14.09%, 6.26%, 5.16%, 4.45%, and 4.05%, respectively. The cumulative contribution rate of the six principal components was 79.71% (Table 1). In 2022, the contribution rates of Cl Composite Index (Cl1-Cl6) for the top six comprehensive evaluation indicators were 59.02%, 7.25%, 5.84%, 5.56%, 4.29%, and 3.76%.

**Table 1:** Coefficient of the comprehensive indexes CIx (comprehensive indexes) and proportion in 2021

Principle factors	Cl_1_	Cl_2_	Cl_3_	Cl_4_	Cl_5_	Cl_6_
Factor weight	0.57	0.18	0.08	0.06	0.06	0.05
Eigenvalue	12.80	3.94	1.75	1.45	1.25	1.13
Coutributive ratio (%)	45.70	14.09	6.26	5.16	4.45	4.05
Cumulative contribution rate (%)	45.70	59.79	66.04	71.21	75.66	79.71
Eigenvector						
Leaf area	0.80	-0.39	0.09	0.05	0.00	-0.11
Plant height	0.54	-0.35	0.03	0.03	-0.02	-0.16
SPAD	0.08	0.02	0.32	-0.08	0.75	-0.21
Canopy temperature	0.24	0.11	0.35	0.18	0.62	-0.04
Leaf water potential;	0.87	-0.29	-0.16	-0.14	0.01	-0.04
Relative water content	0.82	-0.32	-0.25	-0.12	0.10	0.01
Root dry weight;	0.90	-0.15	0.22	-0.15	-0.12	-0.01
Fresh root/shoot ratio	0.44	-0.44	0.29	-0.07	-0.25	-0.15
Dry root/shoot ratio	-0.52	0.14	0.18	0.12	-0.22	-0.47
Water loss rate of shoot	0.05	-0.10	0.31	-0.38	-0.06	0.58
Water loss rate of root	-0.28	0.13	-0.04	-0.35	0.24	0.48
Total root length	0.69	0.51	0.35	-0.18	-0.15	0.01
Projection area	0.74	0.54	0.10	-0.14	-0.02	-0.09
Surface area	0.83	0.45	0.18	-0.14	-0.05	-0.05
Average diameter	-0.90	0.29	-0.12	0.07	0.05	0.03
Average volume	0.85	0.38	0.24	-0.17	-0.09	-0.02
Average lateral root emergence angle	0.90	-0.34	-0.09	-0.03	0.05	-0.05
Average lateral root tip angle	0.86	-0.36	-0.04	-0.08	0.03	-0.01
Average length—all roots	0.47	0.22	0.32	0.58	-0.14	0.31
Average length—all lateral roots	0.77	-0.24	-0.08	0.05	-0.11	0.09
Lateral root count	0.66	0.10	-0.30	0.45	0.03	0.28
Maximum width	0.81	-0.47	0.02	0.02	0.05	-0.01
Maximum depth	0.69	0.41	-0.15	0.47	0.04	0.13
Width/depth ratio	0.83	-0.35	-0.12	0.21	0.13	0.06
Specific root length	0.55	0.72	0.08	-0.14	-0.09	-0.04
Specific root surface area	0.59	0.66	-0.27	-0.01	0.03	-0.10
Specific root volume	0.65	0.62	-0.25	-0.01	0.08	-0.11
Root tissue density	-0.31	-0.17	0.74	0.33	-0.09	0.05

**Table 2:** Coefficient of the comprehensive indexes CIx (comprehensive indexes) and proportion in 2022

Principle factors	Cl_1_	Cl_2_	Cl_3_	Cl_4_	Cl_5_	Cl_6_
Factor weight	0.69	0.08	0.07	0.06	0.05	0.04
Eigenvalue	16.53	2.03	1.63	1.56	1.20	1.05
Coutributive ratio (%)	59.02	7.25	5.84	5.56	4.29	3.76
Cumulative contribution rate (%)	59.02	66.27	72.11	77.67	81.96	85.72
Eigenvector						
Leaf area	0.96	0.10	0.00	-0.01	0.04	0.08
Plant height	0.94	-0.22	-0.04	-0.03	0.10	0.00
SPAD	0.79	-0.39	-0.02	-0.13	0.02	-0.18
Canopy temperature	0.95	-0.19	-0.05	-0.01	0.05	-0.01
Leaf water potential;	0.80	-0.38	0.28	-0.05	-0.01	0.06
Relative water content	0.71	-0.39	0.28	0.20	-0.10	0.09
Root dry weight;	0.95	0.20	-0.08	0.10	-0.02	0.14
Fresh root/shoot ratio	0.26	0.18	0.04	-0.65	0.37	-0.02
Dry root/shoot ratio	0.55	0.35	-0.35	0.25	0.06	-0.46
Water loss rate of shoot	0.28	0.35	-0.72	-0.04	0.13	0.12
Water loss rate of root	-0.16	-0.05	-0.19	0.81	-0.13	0.08
Total root length	0.75	0.42	-0.01	-0.15	0.10	0.13
Projection area	0.91	0.16	-0.03	0.11	-0.04	0.18
Surface area	0.62	0.49	0.34	-0.18	-0.23	-0.21
Average diameter	0.47	0.14	0.16	0.37	0.51	-0.53
Average volume	0.67	0.34	0.11	-0.12	-0.09	0.03
Average lateral root emergence angle	0.95	0.10	0.02	0.09	-0.05	0.10
Average lateral root tip angle	0.97	-0.16	-0.02	0.01	0.02	0.04
Average length—all roots	0.97	-0.01	-0.07	0.05	0.04	0.10
Average length—all lateral roots	0.98	0.04	-0.04	0.08	-0.01	0.10
Lateral root count	0.94	0.13	-0.03	0.11	-0.05	0.15
Maximum width	0.91	-0.30	-0.08	-0.09	-0.02	-0.08
Maximum depth	0.96	-0.11	0.04	0.03	-0.04	0.05
Width/depth ratio	0.85	-0.41	-0.09	-0.13	-0.06	-0.14
Specific root length	0.85	0.25	0.02	0.06	0.05	0.20
Specific root surface area	-0.15	0.13	0.62	0.28	0.59	0.21
Specific root volume	0.48	0.33	0.42	0.03	-0.52	-0.36
Root tissue density	-0.42	0.31	0.22	0.08	-0.19	0.30

The cumulative contribution rate of the six principal components was 85.72%. The two years of experimental data indicated that by transforming the original 28 single traits into six independent composite indicators, i.e., six principal factors, most of the information could be covered (Table 2). In 2021, root volume, surface area, average lateral root emergence angle, average diameter, total root length, and root dry weight had higher load coefficients. However, in 2022, average length—all lateral roots, average lateral root tip angle, root dry weight, average length—all roots, canopy temperature, and leaf area had higher load coefficients. These traits primarily reflected cotton's root traits and canopy parameters.

### Comprehensive evaluation and screening of drought tolerance traits

Subordinate function values of 80 variety composite indicators were calculated according to formula (3) (Tables 3 and 4). In this principal component, higher Cl values indicate greater drought tolerance, while lower values indicate weaker drought tolerance. Based on the contribution rates of each composite indicator, indicator weights were calculated using formula (4).

**Table 3:** The membership function value of each cultivars in 2021

U(X_i_)	U(X_1_)	U(X_2_)	U(X_3_)	U(X_4_)	U(X_5_)	U(X_6_)	U(X_i_)	U(X_1_)	U(X_2_)	U(X_3_)	U(X_4_)	U(X_5_)	U(X_6_)
1	0.04	0.30	0.77	0.56	0.30	0.00	41	0.12	0.34	0.36	0.42	0.49	0.59
2	0.12	0.45	0.66	0.67	0.27	0.29	42	0.14	0.23	0.67	0.37	0.37	0.15
3	0.12	0.46	0.68	0.63	0.28	0.27	43	0.09	0.47	0.68	0.75	0.14	0.23
4	0.17	0.49	0.74	0.74	0.25	0.36	44	0.13	0.40	0.52	0.57	0.40	0.40
5	0.18	0.55	0.70	0.73	0.26	0.41	45	0.27	0.43	0.65	0.63	0.17	0.23
6	0.23	0.64	0.70	0.76	0.24	0.48	46	0.13	0.68	0.69	0.85	0.20	0.29
7	0.17	0.54	0.70	0.78	0.28	0.41	47	0.00	0.30	0.00	0.25	0.69	0.66
8	0.14	0.49	0.66	0.64	0.28	0.31	48	0.02	0.00	0.22	0.48	0.39	0.46
9	0.17	0.50	0.64	0.62	0.28	0.40	49	0.06	0.25	0.65	0.47	0.27	0.18
10	0.14	0.42	0.57	0.64	0.19	0.38	50	0.02	0.26	0.63	0.53	0.26	0.19
11	0.12	0.47	0.62	0.59	0.30	0.32	51	0.10	0.06	0.72	0.00	0.00	0.01
12	0.04	0.35	0.51	0.56	0.25	0.27	52	0.03	0.39	0.68	0.57	0.27	0.18
13	0.10	0.37	0.63	0.58	0.30	0.27	53	0.10	0.09	0.61	0.24	0.50	0.16
14	0.22	0.61	0.80	0.78	0.46	0.35	54	0.04	0.50	0.44	0.70	0.15	0.37
15	0.19	0.59	0.76	0.74	0.26	0.36	55	0.59	0.20	0.76	0.14	0.59	0.25
16	0.20	0.64	0.71	0.75	0.20	0.38	56	0.06	0.94	0.73	0.87	0.00	0.23
17	0.05	0.05	0.63	0.36	0.34	0.21	57	0.15	0.16	0.56	0.14	0.50	0.40
18	0.17	0.53	0.66	0.72	0.25	0.37	58	0.18	0.55	0.68	0.67	0.16	0.31
19	0.12	0.45	0.65	0.58	0.29	0.29	59	0.07	0.46	0.69	0.69	0.21	0.29
20	0.21	0.65	0.63	0.78	0.24	0.44	60	0.12	0.26	0.70	0.55	0.37	0.26
21	0.50	0.79	0.87	0.86	0.57	0.56	61	0.20	0.77	0.61	0.64	0.18	0.40
22	0.09	0.37	0.68	0.54	0.30	0.22	62	0.08	0.53	0.65	0.67	0.21	0.36
23	0.16	0.49	0.62	0.59	0.33	0.47	63	0.20	0.13	0.69	0.17	0.46	0.23
24	0.34	0.77	0.74	0.79	0.36	0.46	64	0.20	1.00	0.65	0.81	0.03	0.26
25	0.17	0.59	0.66	0.75	0.27	0.41	65	0.05	0.33	0.36	0.55	0.33	0.44
26	0.12	0.39	0.62	0.60	0.32	0.38	66	0.09	0.36	0.64	0.68	0.28	0.21
27	0.21	0.65	0.65	0.63	0.32	0.43	67	0.00	0.09	0.66	0.53	0.27	0.09
28	0.14	0.48	0.66	0.66	0.24	0.29	68	-	-	-	-	-	-
29	0.22	0.64	0.88	0.79	0.27	0.27	69	0.01	0.12	0.37	0.30	0.38	0.24
30	0.13	0.57	0.66	0.68	0.28	0.41	70	0.10	0.37	0.70	0.55	0.27	0.20
31	0.16	0.52	0.68	0.68	0.28	0.37	71	1.00	0.91	0.93	1.00	1.00	0.57
32	0.10	0.36	0.69	0.61	0.33	0.30	72	0.11	0.44	0.66	0.63	0.33	0.37
33	0.09	0.10	0.62	0.21	0.40	0.17	73	0.04	0.16	0.48	0.31	0.32	0.26
34	0.14	0.58	0.61	0.64	0.20	0.46	74	0.07	0.34	0.69	0.50	0.26	0.20
35	0.14	0.41	0.68	0.45	0.32	0.39	75	0.13	0.11	0.77	0.09	0.28	0.04
36	0.28	0.24	0.57	0.53	0.43	0.23	76	0.12	0.44	0.65	0.58	0.27	0.31
37	0.50	0.94	0.44	0.81	0.49	1.00	77	0.30	0.72	0.72	0.79	0.32	0.60
38	0.37	0.75	1.00	0.73	0.20	0.21	78	0.33	0.80	0.87	0.86	0.28	0.41
39	0.18	0.61	0.63	0.80	0.31	0.55	79	0.05	0.38	0.67	0.57	0.31	0.21
40	0.09	0.79	0.57	0.68	0.21	0.37	80	0.21	0.80	0.78	0.73	0.32	0.32

Note: 1, Jifeng 554; 2, Jifeng 103; 3, Jifeng 522; 4, Jifeng 908; 5, Jifeng 914; 6, Jifeng 1982; 7, Jifeng 4; 8, 7886; 9, Cangmian 268; 10, Jimian 315; 11, Han 218; 12, Hannong 12; 13, Han 8266; 14, Han 258; 15, Han 686; 16, YM111; 17, Nongda KZ05; 18, Nongdamian 10; 19, Nongdamian 12; 20, Lumianyan 28; 21, Xuzhou 1818; 22, Zhongmiansuo 41; 23, Shandongxiamian11-42; 24, Zhongmiansuo 12; 25, Yumian 19; 26, Ejing 1; 27, Zhongmiansuo 35; 28, Zhongmiansuo 60; 29, Xinshi 71143; 30, Xinza 15; 31, Xinshi 17; 32, GK39; 33, 0 shi; 34, Zhongmiansuo 94A915; 35, Lumianyan 36; 36, DP33B; 37, Guoxinmian01; 38, Guoxinmian02; 39, Guoxinmian03; 40, Guoxinmian05; 41, Hanwu 216; 42, Zhongmian 100; 43, Zhongmiansuo 79; 44, Cangmian 666; 45, Han 6203; 46, Shikang 126; 47, Cang 198; 48, Ji 228; 49, Guoxinmian 9; 50, K836; 51, Lumian 522; 52, Lumian 5172; 53, K638; 54, Guoxin 4; 55, Jifeng1187; 56, Jifeng 1458; 57, Jifeng 103; 58, Jifeng 914; 59, Jifeng 965; 60, MH335223; 61, Guoxinmian 11; 62, Zhongmiansuo 17; 63, Chunbeibao; 64, Zhongmiansuo 60;65, CG3020-3; 66, Jimian 2016; 67, Ji 1518; 68, Jihang 8; 69, Jimian 262; 70, Ji 178; 71, Ji 172; 72, Yuzaomian 9110; 73, Dexiamian 1; 74, Jicai 6913; 75, Zhongmiansuo 23; 76, Zhongmiansuo 50; 77, Ji668; 78, Zhibao 86–1; 79, Jimian 958; 80, Jifeng 1271

**Table 4:** The membership function value of each cultivars in 2022

U(X_i_)	U(X_1_)	U(X_2_)	U(X_3_)	U(X_4_)	U(X_5_)	U(X_6_)	U(X_i_)	U(X_1_)	U(X_2_)	U(X_3_)	U(X_4_)	U(X_5_)	U(X_6_)
1	0.35	0.77	0.48	0.29	0.28	0.77	41	0.38	0.77	0.33	0.26	0.16	0.92
2	0.44	0.88	0.61	0.37	0.39	0.77	42	0.32	0.84	0.35	0.29	0.16	0.92
3	0.44	0.88	0.55	0.37	0.37	0.80	43	0.49	0.86	0.72	0.50	0.50	0.65
4	0.51	0.88	0.65	0.48	0.48	0.69	44	0.31	0.84	0.52	0.31	0.29	0.84
5	0.56	0.87	0.65	0.47	0.48	0.70	45	0.56	0.88	0.65	0.52	0.35	0.71
6	0.59	0.93	0.77	0.57	0.59	0.60	46	0.63	0.66	0.92	0.76	0.89	0.26
7	0.64	0.90	0.73	0.53	0.58	0.61	47	0.33	0.60	0.50	0.32	0.11	0.88
8	0.49	0.83	0.63	0.45	0.40	0.71	48	0.20	0.88	0.21	0.10	0.07	0.94
9	0.53	0.89	0.55	0.40	0.37	0.78	49	0.29	0.81	0.40	0.20	0.18	0.91
10	0.46	0.86	0.63	0.35	0.40	0.75	50	0.32	0.95	0.47	0.27	0.28	0.86
11	0.41	0.87	0.58	0.32	0.35	0.79	51	0.28	0.83	0.37	0.16	0.14	0.92
12	0.37	0.87	0.44	0.29	0.25	0.88	52	0.31	0.91	0.53	0.31	0.31	0.83
13	0.37	0.84	0.51	0.30	0.28	0.82	53	0.23	0.95	0.38	0.17	0.11	0.93
14	0.57	0.86	0.72	0.54	0.54	0.63	54	0.46	0.89	0.66	0.41	0.47	0.68
15	0.52	0.69	0.70	0.49	0.50	0.67	55	0.43	0.74	0.35	0.21	0.08	0.95
16	0.62	0.75	0.79	0.59	0.58	0.57	56	0.73	0.78	0.89	0.88	0.94	0.00
17	0.28	0.78	0.41	0.28	0.20	0.89	57	0.27	0.64	0.12	0.08	0.00	1.00
18	0.51	0.82	0.58	0.49	0.46	0.74	58	0.64	0.94	0.73	0.54	0.44	0.68
19	0.54	0.88	0.49	0.38	0.31	0.83	59	0.52	0.89	0.69	0.44	0.48	0.66
20	0.49	0.89	0.74	0.61	0.62	0.64	60	0.38	0.87	0.51	0.31	0.27	0.85
21	0.73	0.91	0.87	0.86	0.82	0.50	61	0.56	0.90	0.68	0.52	0.39	0.72
22	0.27	0.87	0.49	0.30	0.25	0.86	62	0.44	0.86	0.60	0.35	0.40	0.81
23	0.41	0.86	0.58	0.32	0.35	0.80	63	0.38	0.00	0.34	0.00	0.06	0.97
24	0.72	0.82	0.83	0.75	0.80	0.41	64	0.55	0.95	0.77	0.64	0.67	0.51
25	0.65	0.85	0.71	0.59	0.54	0.67	65	0.29	0.85	0.39	0.21	0.19	0.88
26	0.45	0.89	0.56	0.34	0.34	0.78	66	0.45	0.83	0.61	0.42	0.42	0.74
27	0.45	0.42	0.65	0.40	0.42	0.74	67	0.40	0.79	0.48	0.19	0.22	0.87
28	0.61	0.89	0.63	0.44	0.33	0.52	68	-	-	-	-	-	-
29	0.72	0.91	0.81	0.84	0.76	0.47	69	0.25	0.46	0.37	0.14	0.14	0.93
30	0.50	0.87	0.65	0.45	0.46	0.69	70	0.36	0.87	0.50	0.34	0.27	0.84
31	0.68	0.88	0.62	0.55	0.49	0.69	71	1.00	0.90	1.00	1.00	0.93	0.11
32	0.32	0.88	0.53	0.37	0.33	0.82	72	0.41	0.82	0.54	0.37	0.30	0.85
33	0.40	0.87	0.43	0.25	0.20	0.89	73	0.34	0.83	0.37	0.24	0.20	0.86
34	0.49	0.88	0.70	0.54	0.49	0.64	74	0.31	0.83	0.41	0.24	0.21	0.89
35	0.37	0.84	0.47	0.27	0.24	0.75	75	0.00	0.54	0.00	0.05	0.08	0.99
36	0.36	0.71	0.42	0.29	0.20	0.92	76	0.43	0.87	0.57	0.36	0.35	0.72
37	0.70	0.93	0.78	0.74	0.62	0.62	77	0.59	0.95	0.76	0.64	0.62	0.65
38	0.60	0.97	0.88	0.93	1.00	0.41	78	0.63	1.00	0.79	0.71	0.80	0.69
39	0.95	0.92	0.65	0.78	0.57	0.33	79	0.30	0.60	0.50	0.24	0.23	0.71
40	0.43	0.89	0.68	0.48	0.50	0.69	80	0.40	0.89	0.65	0.37	0.42	0.78

Note: 1, Jifeng 554; 2, Jifeng 103; 3, Jifeng 522; 4, Jifeng 908; 5, Jifeng 914; 6, Jifeng 1982; 7, Jifeng 4; 8, 7886; 9, Cangmian 268; 10, Jimian 315; 11, Han 218; 12, Hannong 12; 13, Han 8266; 14, Han 258; 15, Han 686; 16, YM111; 17, Nongda KZ05; 18, Nongdamian 10; 19, Nongdamian 12; 20, Lumianyan 28; 21, Xuzhou 1818; 22, Zhongmiansuo 41; 23, Shandongxiamian11-42; 24, Zhongmiansuo 12; 25, Yumian 19; 26, Ejing 1; 27, Zhongmiansuo 35; 28, Zhongmiansuo 60; 29, Xinshi 71143; 30, Xinza 15; 31, Xinshi 17; 32, GK39; 33, 0 shi; 34, Zhongmiansuo 94A915; 35, Lumianyan 36; 36, DP33B; 37, Guoxinmian01; 38, Guoxinmian02; 39, Guoxinmian03; 40, Guoxinmian05; 41, Hanwu 216; 42, Zhongmian 100; 43, Zhongmiansuo 79; 44, Cangmian 666; 45, Han 6203; 46, Shikang 126; 47, Cang 198; 48, Ji 228; 49, Guoxinmian 9; 50, K836; 51, Lumian 522; 52, Lumian 5172; 53, K638; 54, Guoxin 4; 55, Jifeng1187; 56, Jifeng 1458; 57, Jifeng 103; 58, Jifeng 914; 59, Jifeng 965; 60, MH335223; 61, Guoxinmian 11; 62, Zhongmiansuo 17; 63, Chunbeibao; 64, Zhongmiansuo 60;65, CG3020-3; 66, Jimian 2016; 67, Ji 1518; 68, Jihang 8; 69, Jimian 262; 70, Ji 178; 71, Ji 172; 72, Yuzaomian 9110; 73, Dexiamian 1; 74, Jicai 6913; 75, Zhongmiansuo 23; 76, Zhongmiansuo 50; 77, Ji668; 78, Zhibao 86–1; 79, Jimian 958; 80, Jifeng 1271

After calculation, the weights of the six composite indicators in 2021 were 0.57, 0.18, 0.08, 0.06, 0.06, and 0.05, respectively (Table 1). In 2022, the weights of the six composite indicators were 0.69, 0.08, 0.07, 0.06, 0.05, and 0.04, respectively (Table 2). Equation (5) was used to calculate the drought tolerance of different cotton cultivars (Tables 3 and 4), and the drought tolerance of different cotton cultivars was ranked according to the D-value (Tables 5 and 6). A smaller D-value indicates poorer drought tolerance, whereas greater drought tolerance corresponds to larger D-values (Tables 5 and 6).

**Table 5:** D-value of each variety in 2021

Variety	D-value	Rank	Variety	D-value	Rank	Variety	D-value	Rank	Variety	D-value	Rank
Jifeng 554	0.19	65	Xuzhou 1818	0.61	3	Hanwu 216	0.24	52	Guoxinmian 11	0.37	15
Jifeng 103	0.27	42	Zhongmiansuo 41	0.23	58	Zhongmian 100	0.22	59	Zhongmiansuo 17	0.26	47
Jifeng 522	0.28	38	Shandongxiamian11-42	0.31	35	Zhongmiansuo 79	0.25	49	Chunbeibao	0.24	56
Jifeng 908	0.32	27	Zhongmiansuo 12	0.48	7	Cangmian 666	0.27	46	Zhongmiansuo 60	0.41	9
Jifeng 914	0.34	22	Yumian 19	0.34	21	Han 6203	0.34	20	CG3020-3	0.19	67
Jifeng 1982	0.39	13	Ejing 1	0.26	48	Shikang 126	0.33	25	Jimian 2016	0.24	57
Jifeng 4	0.34	23	Zhongmiansuo 35	0.36	16	Cang 198	0.14	73	Ji 1518	0.12	77
7886	0.29	36	Zhongmiansuo 60	0.29	37	Ji 228	0.1	79	Jihang 8	-	-
Cangmian 268	0.31	31	Xinshi 71143	0.39	11	Guoxinmian 9	0.18	68	Jimian 262	0.11	78
Jimian 315	0.27	45	Xinza 15	0.31	34	K836	0.16	71	Ji 178	0.24	53
Han 218	0.27	41	Xinshi 17	0.32	30	Lumian 522	0.12	76	Ji 172	0.96	1
Hannong 12	0.19	66	GK39	0.25	50	Lumian 5172	0.2	64	Yuzaomian 9110	0.27	40
Han 8266	0.24	54	0 shi	0.16	72	K638	0.17	70	Dexiamian 1	0.14	75
Han 258	0.39	12	Zhongmiansuo 94A915	0.31	32	Guoxin 4	0.22	60	Jicai 6913	0.21	63
Han 686	0.36	19	Lumianyan 36	0.27	39	Jifeng1187	0.49	6	Zhongmiansuo 23	0.18	69
YM111	0.36	17	DP33B	0.32	29	Jifeng 1458	0.33	24	Zhongmiansuo 50	0.27	44
Nongda KZ05	0.14	74	Guoxinmian01	0.62	2	Jifeng 103	0.22	61	Ji668	0.46	8
Nongdamian 10	0.32	26	Guoxinmian02	0.49	4	Jifeng 914	0.32	28	Zhibao 86–1	0.49	5
Nongdamian 12	0.27	43	Guoxinmian03	0.36	18	Jifeng 965	0.25	51	Jimian 958	0.22	62
Lumianyan 28	0.37	14	Guoxinmian05	0.31	33	MH335223	0.24	55	Jifeng 1271	0.4	10

**Table 6:** D-value of each variety in 2022

Variety	D-value	Rank	Variety	D-value	Rank	Variety	D-value	Rank	Variety	D-value	Rank
Jifeng 554	0.4	60	Xuzhou 1818	0.75	3	Hanwu 216	0.41	57	Guoxinmian 11	0.59	21
Jifeng 103	0.49	41	Zhongmiansuo 41	0.37	68	Zhongmian 100	0.38	65	Zhongmiansuo 17	0.5	39
Jifeng 522	0.49	42	Shandongxiamian11-42	0.46	48	Zhongmiansuo 79	0.55	31	Chunbeibao	0.33	74
Jifeng 908	0.55	28	Zhongmiansuo 12	0.73	6	Cangmian 666	0.39	64	Zhongmiansuo 60	0.61	19
Jifeng 914	0.59	23	Yumian 19	0.67	11	Han 6203	0.59	22	CG3020-3	0.36	69
Jifeng 1982	0.63	17	Ejing 1	0.49	40	Shikang 126	0.66	12	Jimian 2016	0.5	38
Jifeng 4	0.66	13	Zhongmiansuo 35	0.47	45	Cang 198	0.38	67	Ji 1518	0.44	52
7886	0.53	34	Zhongmiansuo 60	0.61	18	Ji 228	0.28	78	Jihang 8	-	-
Cangmian 268	0.56	24	Xinshi 71143	0.74	4	Guoxinmian 9	0.35	70	Jimian 262	0.29	77
Jimian 315	0.51	36	Xinza 15	0.54	33	K836	0.4	61	Ji 178	0.43	53
Han 218	0.47	46	Xinshi 17	0.68	10	Lumian 522	0.34	73	Ji 172	0.95	1
Hannong 12	0.43	54	GK39	0.41	58	Lumian 5172	0.4	62	Yuzaomian 9110	0.47	47
Han 8266	0.43	55	0 shi	0.44	50	K638	0.32	75	Dexiamian 1	0.4	63
Han 258	0.6	20	Zhongmiansuo 94A915	0.55	30	Guoxin 4	0.51	35	Jicai 6913	0.38	66
Han 686	0.55	29	Lumianyan 36	0.42	56	Jifeng1187	0.44	49	Zhongmiansuo 23	0.1	79
YM111	0.64	16	DP33B	0.4	59	Jifeng 1458	0.73	5	Zhongmiansuo 50	0.48	43
Nongda KZ05	0.35	71	Guoxinmian01	0.72	7	Jifeng 103	0.3	76	Ji668	0.64	15
Nongdamian 10	0.55	32	Guoxinmian02	0.68	9	Jifeng 914	0.66	14	Zhibao 86–1	0.69	8
Nongdamian 12	0.56	25	Guoxinmian03	0.87	2	Jifeng 965	0.56	27	Jimian 958	0.35	72
Lumianyan 28	0.56	26	Guoxinmian05	0.5	37	MH335223	0.44	51	Jifeng 1271	0.47	44

**Fig. 3:**
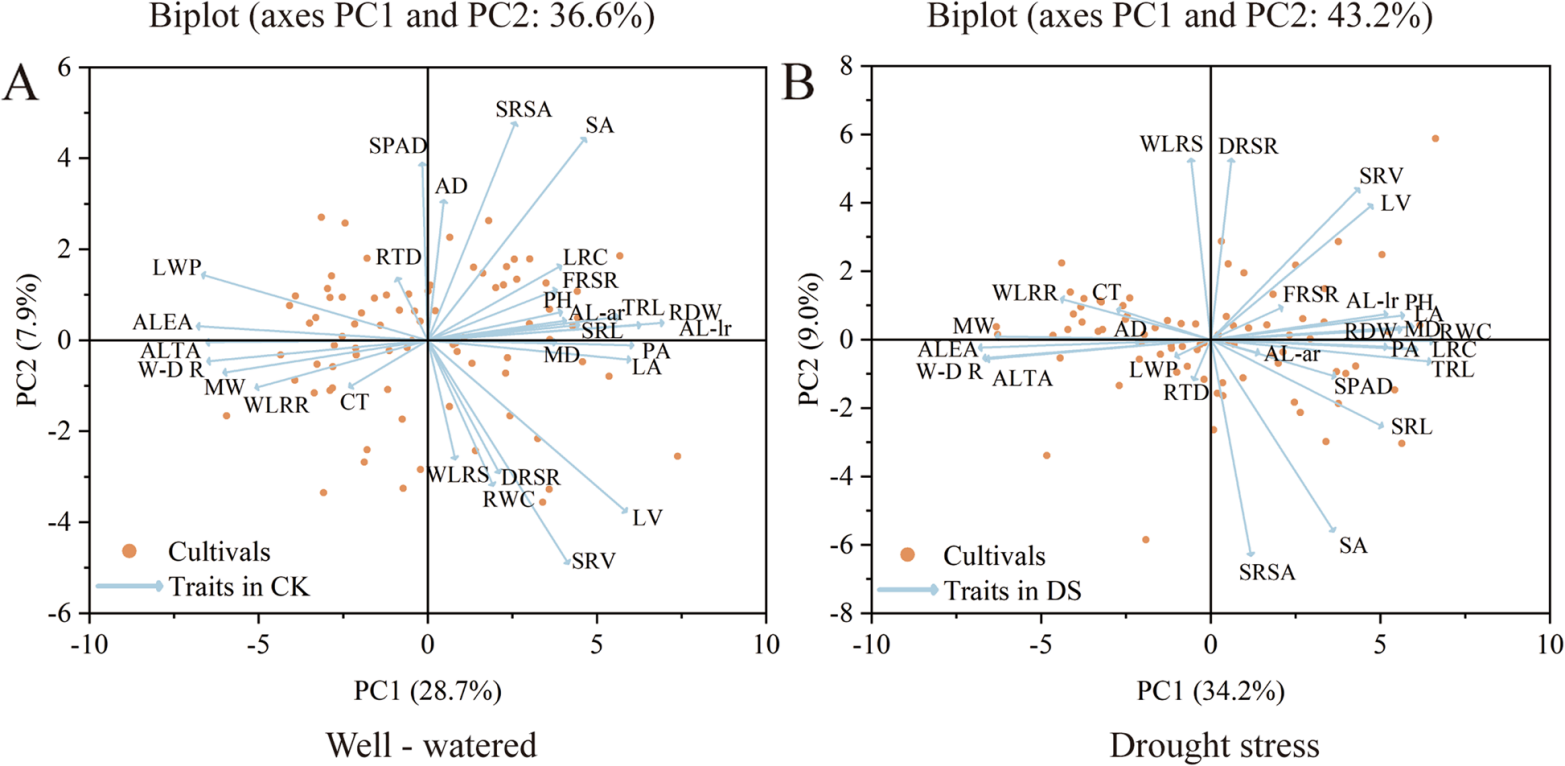
Biplot analysis of 80 cotton cultivars and 28 different traits under drought stress in 2021 (**A**) and 2022 (**B**). LA, leaf area; PH, plant height; CT, canopy temperature; LWP, leaf water potential; RWC, relative water content; RDW, root dry weight; FRSR, fresh root/shoot ratio; DRSR, dry root/shoot ratio; WLRS, water loss rate of shoot; WLRR, water loss rate of root; TRL, total root length; PA, projection area; SA, surface area; AD, average diameter; AV, average volume; ALEA, average lateral root emergence angle; ALTA, average lateral root tip angle; AL-ar, average length—all roots; AL-lr, average length—all lateral roots; LRC, lateral root count; MW, maximum width; MD, maximum depth; W/D R, width/depth ratio; SRL, specific root length; SRSA, specific root surface area; SRV, specific root volume; RTD, root tissue density

We used PCA to evaluate 28 traits under well-watered and drought stress conditions (Fig. 3). In both well-watered and drought stress conditions, the first two principal components contributed 36.6% and 43.2%, respectively (Fig. 3). Specifically, under well-watered conditions, PC1 accounted for 28.7%, while PC2 explained 7.9% of the variance. PC1 was primarily characterized by SPAD, average diameter, canopy temperature, and water loss rate of the shoot, whereas PC2 predominantly featured average length—all lateral roots, projected area, average lateral root emergence angle, and average lateral root tip angle. Conversely, under drought stress, PC1 explained 34.2%, and PC2 explained 9.0%. PC1 was principally represented by water loss rate of the shoot, dry root/shoot ratio, root tissue density, and specific root surface area, while PC2 mainly showcased root width, average lateral root emergence angle, lateral root count, and maximum depth.

### Comprehensive evaluation of drought tolerant cultivars

**Fig. 4:**
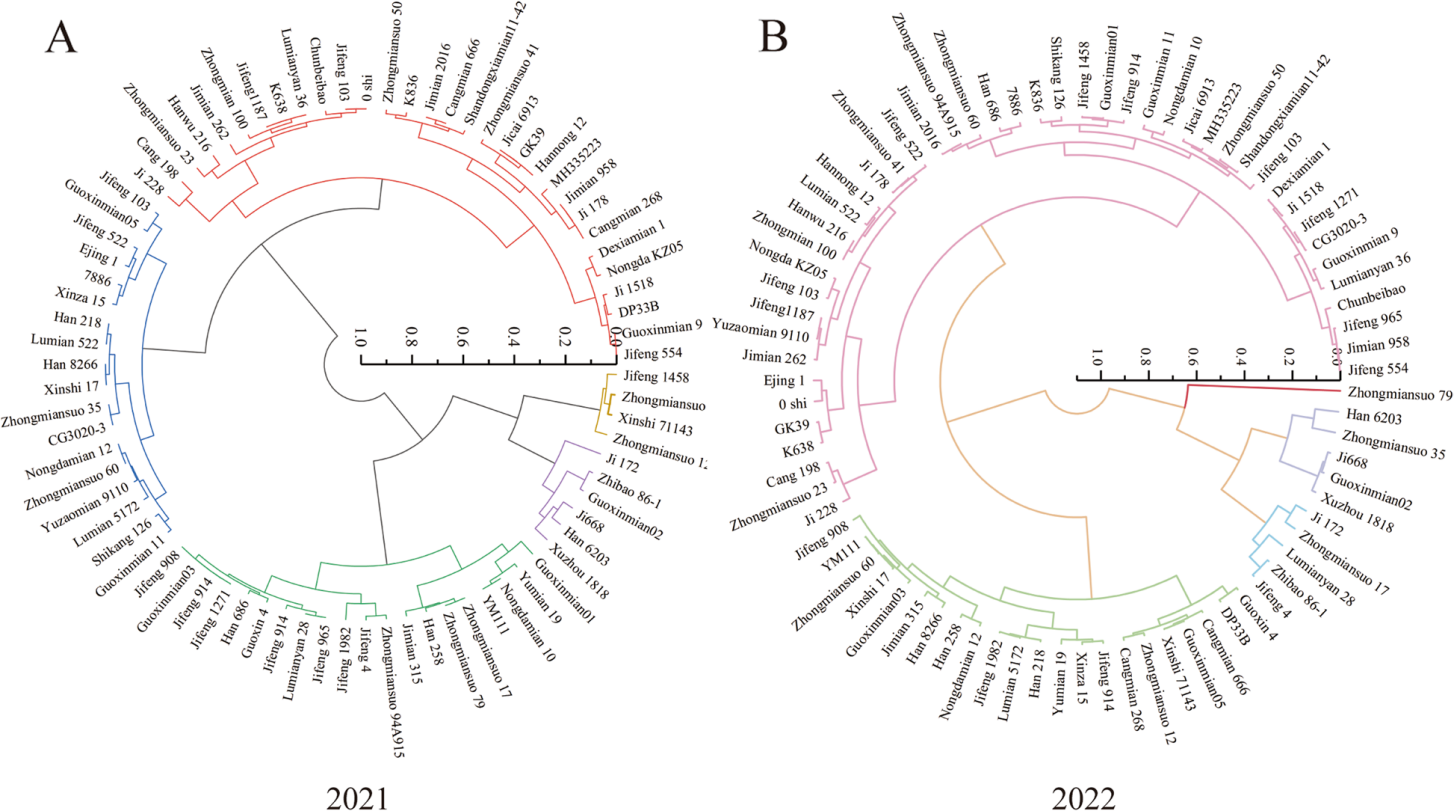
Systematic cluster analyses based on D-values of 80 cotton cultivars were carried out in 2021 (**A**) and 2022 (**B**)

In this investigation, we employed the Euclidean distance flattening method and systematic cluster analysis were used to classify 80 cotton cultivars into five categories based on D-values, including drought-tolerant cultivars, relatively drought-tolerant cultivars, intermediate cultivars, relatively drought-sensitive cultivars, and drought-sensitive cultivars. Our two-year field screening experiments revealed that Ji668, Guoxinmian02, Xuzhou 1818, and Han 6203 demonstrated higher D-values, contrasting with Ji 228, Guoxinmian 9, Zhongmiansuo 23, and Hanwu 216, which displayed lower D-values. Consequently, Ji668, Guoxinmian02, Xuzhou 1818, and Han 6203 can be categorized as drought-tolerance cultivars, whereas Ji 228, Guoxinmian 9, Zhongmiansuo 23, and Hanwu 216 fall into the drought-sensitive cultivars (Fig. 4).

### Validation of biomass and yield drought tolerance index

**Fig. 5:**
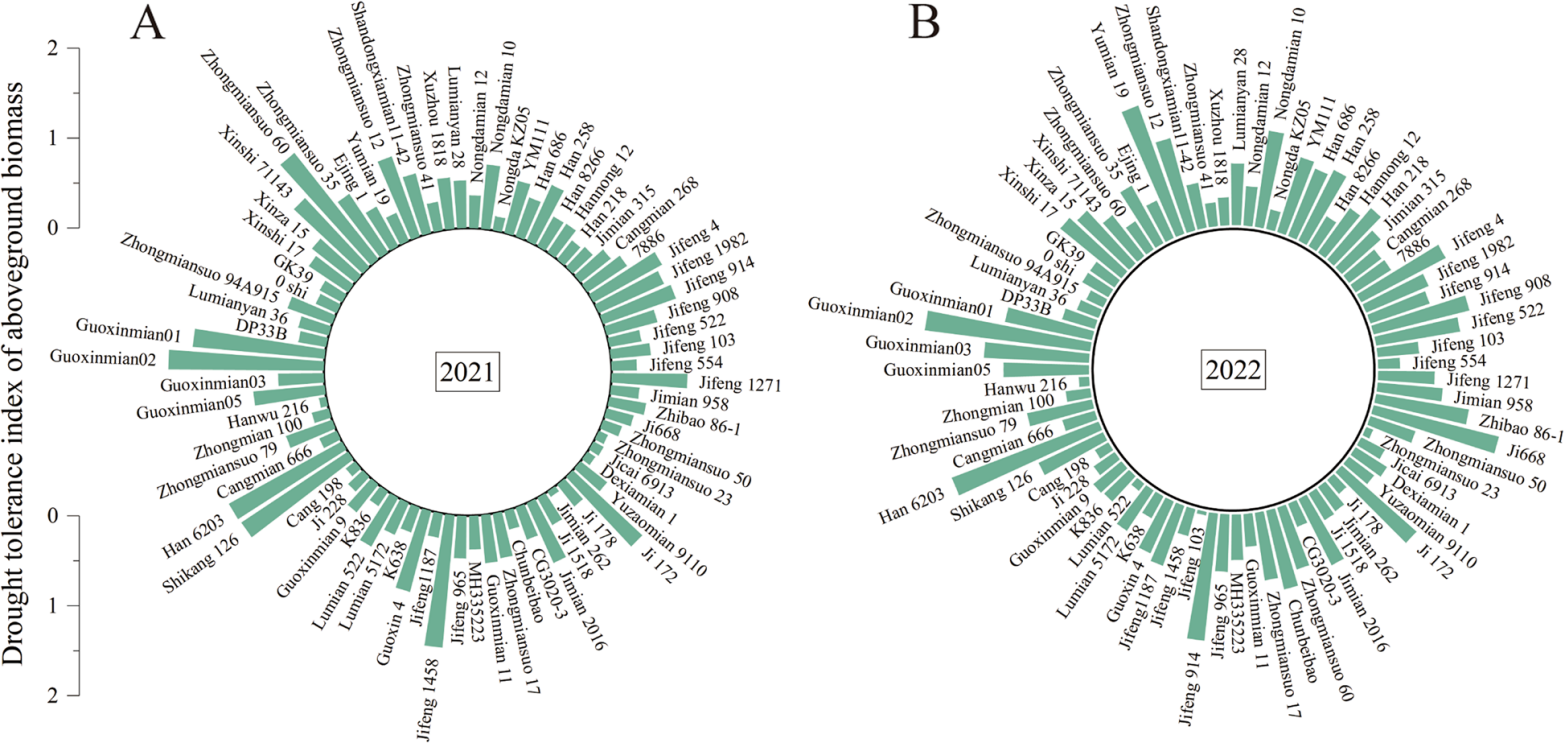
Drought tolerance index of cotton above-ground biomass in 2021 (**A**) and 2022 (**B**)

Under drought stress, there was a significant reduction in both the above-ground biomass and cotton yield (Supplementary Tables 3–6). Nonetheless, drought-tolerance cultivars exhibited considerably higher drought tolerance coefficients in comparison to their drought-sensitive cultivars. Specifically, in 2021, the drought tolerance index of above-ground biomass for drought-tolerance cultivars Ji668, Guoxinmian 02, Xuzhou 1818, and Han 6203 were 0.31, 1.73, 0.57, and 1.43, while for drought-sensitive cultivars Ji 228, Guoxinmian 9, Zhongmiansuo 23, and Hanwu 216 were 0.22, 0.39, 0.11, and 0.08 (Fig. 5A). The trends in 2022 closely mirrored those in 2021 (Fig. 5B).

**Fig. 6:**
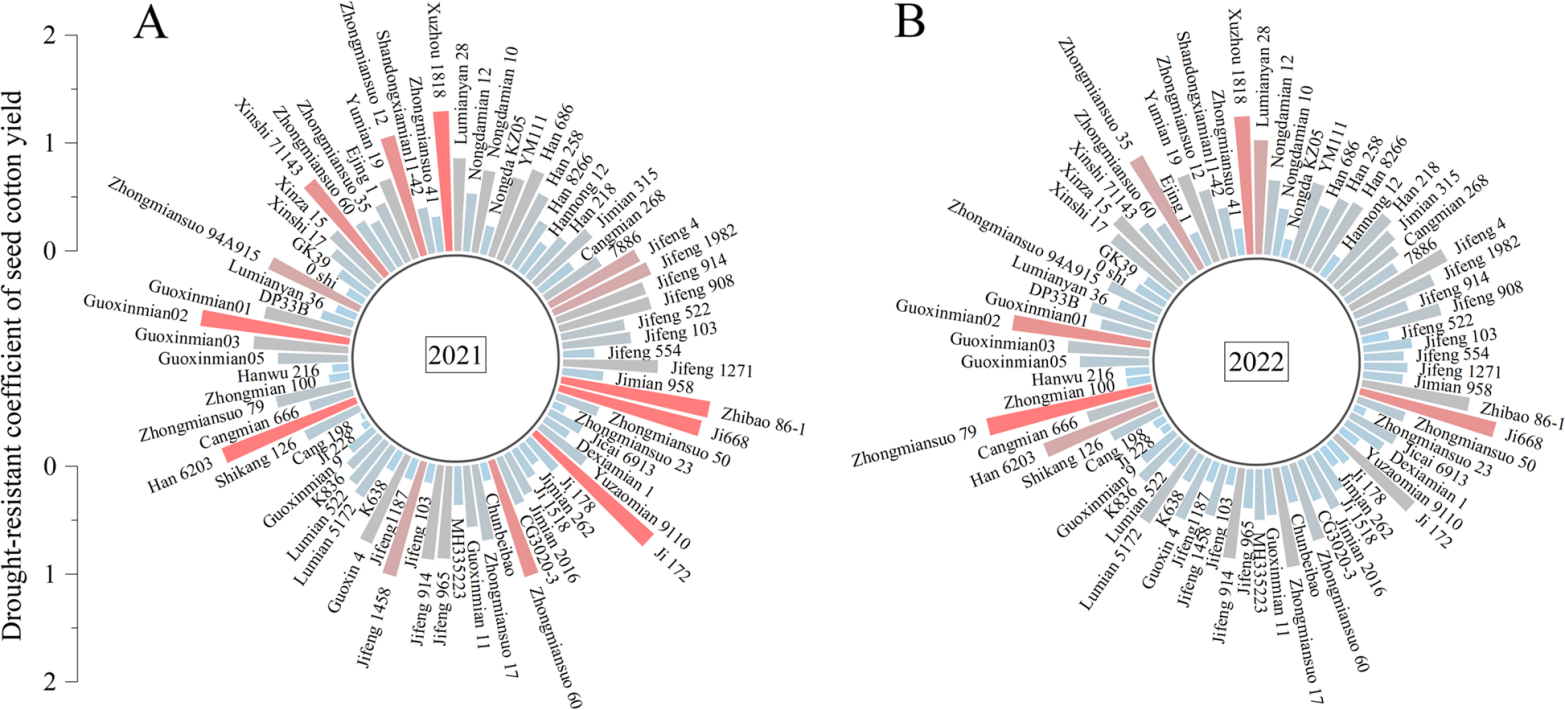
Drought tolerance index of seed cotton yield in 2021 (**A**) and 2022 (**B**)

In 2021, drought tolerance index of yield for drought-tolerance cultivars Ji668, Guoxinmian02, Xuzhou 1818, and Han 6203 were 1.36, 1.39, 1.30, and 1.33, while for drought-sensitive cultivars Ji 228, Guoxinmian 9, Zhongmiansuo 23, and Hanwu 216 were 0.07, 0.29, 0.12, and 0.15 (Fig. 6A). Similar trends were observed in 2022 compared to those in 2021 (Fig. 6B).

## Discussion

In this study, we employed 80 commonly cultivated cotton cultivars in our region to explore their varied responses to drought stress under both well-watered and drought stress conditions. Building upon this investigation, we established an evaluation framework for drought tolerance traits. Additionally, we introduced innovative multivariate statistical methods, including PCA and membership function, alongside a comprehensive screening approach for identifying drought-tolerant cultivars, incorporating the drought tolerance index. These findings aim to serve as a theoretical foundation for the breeding and selection of cotton cultivars exhibiting robust drought tolerance.

### Comprehensive evaluation of drought tolerance

Comprehensive evaluation of drought tolerance provides dependable and objective approaches for investigating crop root screening. The Comprehensive evaluation of drought tolerance method, including PCA, used to assess the drought tolerance of the studied cultivars, presents numerous advantages [27–29]. Firstly, employing dimensionality reduction techniques replaces multiple original variables with a few composite variables, consolidating most information from the initial variables. Secondly, by calculating scores from the comprehensive principal component function, it scientifically evaluates objective phenomena. Thirdly, it emphasizes a comprehensive assessment of the impact of information contribution.

Currently, most studies utilize membership functions and PCA for screening and assessing cultivated cultivars [30]. For example, indoor studies on cotton display the selection of 9 drought-tolerant indicators and 2 cultivars (Desha Cotton No. 1, a drought-tolerant variety, and Yuzao Cotton 9110, a drought-sensitive variety) using the Comprehensive evaluation of drought tolerance method [7]. Similarly, our study validated this analytical approach in the field, classifying 80 cotton cultivars into different drought-tolerant categories and scrutinizing the yield differences among them, further corroborating the Comprehensive evaluation method for assessing drought tolerance under field conditions. Hence, the results obtained through the Comprehensive evaluation of drought tolerance method are credible and scientifically sound.

PCA effectively captures the primary and secondary effects of drought tolerance indicators in cotton and offers a comprehensive assessment of drought tolerance variations among various cotton cultivars. PCA can condense numerous variables into a few underlying factors with minimal loss of information [31]. Employing a single trait or a limited set of traits for assessing drought tolerance in different cultivars is inadequate, necessitating a comprehensive evaluation and screening of multiple traits to prevent redundancy of information. In line with our study, PCA reduced the 28 variables to six underlying factors.

Furthermore, PCA assessed these 28 traits under well-watered and drought stress. The scatter plots within the biplot illustrate the distribution of Factor 1 and Factor 2 for each of the 80 cotton cultivars. PCA provides a holistic view of the primary and secondary effects of the selected drought tolerance indicators in assessing drought tolerance distinctions among the 80 cotton cultivars. In summary, the PCA biplot clarifies the relationships between different indicators under well-watered and drought stess, as well as the contributions of each trait to the principal components.

In general, a comprehensive drought tolerance evaluation approach improves screening results and ensures a more thorough, reliable and accurate evaluation. Such a holistic evaluation approach helps to identify potential drought-tolerant cultivars and provides important insights and guidance for future research and crop breeding efforts aimed at improving drought tolerance.

### Evaluation of drought tolerance of cotton in the field based on Shovelomics

Shovelomics provides dependable and objective approaches for investigating crop root screening. The method of sampling roots in the field is still attracting attention. This approach is advantageous for high-throughput analysis of root samples, enabling the swift collection of root RSA traits in the field, aiding the selection of ideal RSA cultivars in controlled environments. It has been widely adopted in diverse crops, including corn [32, 33], soybean [34], wheat [35], etc. However, despite the widespread application of 'shovel omics' in various crops such as maize, wheat and soybean, its application in the field research of cotton RSA is still a novel method. This study extends the application of this method to the study of root structure and drought tolerance of cotton cultivars under field conditions.

Field and indoor experiments differ in their advantages within research contexts. Indoor experiments face limitations due to factors like container variability and growth substrate differences, potentially resulting in outcomes that don't precisely replicate real field conditions. This lack of authenticity might hinder the representation of genuine environmental conditions, contrasting with field studies, which more accurately mirror actual growth environments. The key advantage of field research lies in its authenticity and realistic setting, closely resembling genuine growth conditions. This aspect enhances the reliability of field research by better mimicking crop behavior and environmental fluctuations during actual growth stages.

Conducting this study in the field offers comprehensive insights into cotton's response to drought. The methods and assessment criteria encompass both root and above-ground traits, providing a more extensive dataset for accurate drought evaluation. In contrast, indoor experiments might struggle to capture these subtle changes and responses within authentic growth environments. Thus, field research enables a better understanding and evaluation of how different cotton cultivars respond to drought in real-world settings, significantly contributing to the selection and assessment of drought-tolerance cultivars.

### Validating drought tolerance index for cotton yield and biomass

Breeding drought-tolerance crop cultivars necessitates a dual consideration of their drought tolerance and capacity for high yields. This is due to drought's tendency to impact crop yield rather than directly causing plant mortality [36]. Therefore, the development of drought-tolerance cultivars requires a balanced approach to ensure their high-yield potential [37]. Achieving this goal involves cultivating strains possessing both drought-tolerance traits and the potential for high yields [38, 39]. However, in the context of drought breeding for crops like maize, some cultivars perform well under drought stress conditions but lack high-yield potential under well-watered conditions [40]. Consequently, the selection of drought-tolerance cultivars necessitates evaluating their performance under both well-watered and drought stress conditions [41].

In our quest to identify cotton cultivars with increased yields under both well-watered and drought stress conditions, we utilized Lan's [42] defined drought index. This index amalgamates the yield under drought stress conditions with that under well-watered. Assessing the drought tolerance index not only gauges crop growth capability under stress but also considers performance in non-stress conditions. Hence, the drought tolerance index facilitates the identification of cultivars excelling under stressful and optimal conditions.

Moreover, our study categorized 80 cotton cultivars into five groups through PCA, membership functions, and cluster analysis. To further validate the scientific robustness of these outcomes, we aimed to verify them using the drought tolerance index concerning yield and above-ground biomass. Within this study, we identified four cultivars Ji668, Guoxinmian 02, Xuzhou 1818, and Han 6203—that displayed heightened drought tolerance in both the drought tolerance index and comprehensive evaluation. These findings are pivotal for selecting drought-tolerance cotton cultivars, as they showcase performance across diverse conditions, furnishing invaluable insights for drought assessment and cultivation.

### The significance of RSA in cotton drought tolerance

Our research underscores the pivotal role of RSA in field, imperative for evaluating how cotton cultivars adapt to drought stress. We observed that the average lateral root emergence angle significantly influences various cultivars under drought stress, aligning with prior maize hybrid studies where high-yielding hybrids showed steeper root angles compared to lower-yielding ones [43].

Moreover, our study highlighted how an excessive root/shoot ratio may impact cotton's drought tolerance, potentially leading to root redundancy without corresponding benefits during drought stress. Therefore, an optimal RSA could serve as a key indicator of cotton's drought tolerance under drought stress conditions. Overall, the variability in RSA characteristics across diverse cotton cultivars underscores distinctions in root system. A focused assessment on RSA when selecting drought-tolerance cultivars can more precisely evaluate a plant's response to drought stress.

## Conclusions

In this study, based on two growing seasons of field trials, we used 80 cotton cultivars commonly grown in our region to investigate their different responses under both well-watered and drought stress conditions. Building on this investigation, we used the comprehensive D-value evaluation method combined with PCA, membership function analysis and systematic cluster analysis. This categorisation method provided new insights into the selection of drought tolerant cultivars. Through PCA, we identified key indicators for evaluating root and canopy temperature, which provide a more comprehensive understanding of the relationships between different traits. Our research highlights the need to consider both root and above-ground traits together when studying plant drought tolerance for a comprehensive understanding of drought tolerance. To identify cotton cultivars with higher yields under well-watered and drought stress conditions, we also used the drought tolerance index to identify four cultivars: Ji668, Guoxinmian 02, Xuzhou 1818 and Han 6203. These lines showed high drought tolerance in both the drought tolerance index and the comprehensive evaluation. The variability of RSA characteristics among cotton cultivars indicates differences in root structure among cultivars, and focusing on RSA can more accurately assess plant response to drought stress when selecting drought-tolerance cultivars This study provides important theoretical and practical support for the comprehensive evaluation of drought tolerance in cotton, providing essential references and guidance for future drought tolerance breeding efforts.

## Materials and methods

### Experimental site

Field experiments were carried out within rain-out shelters at Hebei Agricultural University's Qingyuan Experimental Station (38.85°N, 115.30°E) during 2021 and 2022. Throughout the cotton growing season, which spans from April to October in both 2021 and 2022, the average temperatures were 20.75 °C and 20.96 °C, with total sunshine hours of 2091.4 h and 2199.8 h, respectively. The soil organic matter, total nitrogen, available phosphorus, available potassium, and alkaline hydrolysis nitrogen at 0–20 cm, 20–40 cm and 40–60 cm were 13.83 g·kg^−1^, 0.93 g·kg^−1^, 17.40 mg·kg^−1^, 121.36 mg·kg^−1^, and 69.45 mg·kg^−1^, respectively. Detailed soil bulk density and field water capacity are provided in Supplementary Table 7.

A split-plot experimental design. The main factors were two water treatments: well-watered (WW, 75 ± 5% soil relative water content) and drought stress (DS, 50 ± 5% soil relative water content), with each treatment being replicated three times. The split area is cotton cultivars, a total of 80 representative cultivars in production, most of which are the main cultivars planted in the cotton area of the Yellow River Basin (Supplementary Table 8). Water treatment started from the three-leaf stage and ended at the maturity stage and the soil moisture content was controlled through micro-sprinkler irrigation and soil moisture sensors. Sensoterra (Soil moisture monitoring system, Netherlands) monitored soil moisture content of the 0–30 cm soil layers over the entire growth period (Supplementary Fig. 1).

The cultivars were directly seeded on 24 April 2021 and 2022. The planting density was 90, 000 plants hm^−2^ with a row spacing of 50 cm. Each plot received 450 kg ha^−1^ of compound fertilizer containing 15% N, 15% P_2_O_5_ and 15% K_2_O as base fertilizer. Additionally, 150 kg ha^−1^ urea (46% N) was top dressed at flowering. Pest control, weed control, chemical control and plant pruning were carried out according to DB43/T 286–2006 Cotton cultivation technical code.

### Plant height, leaf area, relative chlorophyll content, and canopy temperature

On July 4, 2021, and July 8, 2022, three representative plants were selected from each plot to measure the individual morphological indices. Plant height (Length of cotyledon node part to main stem growing part) was measured using a ruler. Leaf area was calculated following the length–width coefficient method described by Mao et al. [27]. Leaf relative chlorophyll content and canopy temperature of the functional leaf of the third leaf were measured between 9:00 and 11:00. Leaf relative chlorophyll content of the functional leaf of the third leaf was determined using a SPAD-502 chlorophyll meter (Konica Minolta in Tokyo, Japan). Canopy temperature was measured using a hand-held infrared thermometer (AGRI—THERM II, Model 6110, USA) [44]. During the observations, the sensor probe was positioned 5 cm away from the top third functional leaf, oriented perpendicularly to the leaf's expansion direction, and the probe's height was adjusted as the plant's height increased. Data were recorded when the measurements reached a stable state.

### Leaf water potential and relative water content

On July 4, 2021, and July 8, 2022, three representative plants were randomly selected from each plot. Leaf water potential and relative water content of the functional leaf of the third leaf were measured between 9:00 and 11:00. Leaf water potential was measured using a Model 600 plant pressure chamber (PMS Inc., USA). Relative water content was assessed using the gravimetric method. The fresh weight of the cut cotton leaves was immediately measured using an analytical balance. Leaf samples were then transported to the laboratory and placed in deionized water for 8–12 h to determine the turgid weight. Subsequently, the samples were placed in an oven, heated to 105 °C, and dried to a constant weight at 80 °C to obtain the dry weight of the leaves.

### Root sampling and analysis

*Root sampling* On 2 July 2021 and 5 July 2022, using the "Shovelomics" method to systematically collect RSA [45]. Three representative cotton plants were randomly selected from each plot. Standard shovels were utilized for excavation, extracting soil blocks measuring 20 cm × 55 cm × 40 cm (plant spacing × row spacing × depth) centered around the plant's root system. The excavated root systems were gently agitated to remove most adhering soil. Subsequently, the root systems were immersed in a 0.5% mild detergent solution to eliminate residual soil. In the next step, remaining soil particles and detergent were removed from the root systems through vigorous rinsing under low pressure. This process yielded clean root samples for further analysis.

*Imaging tent and camera information* The entire setup was computer-controlled for image acquisition. Uniform and consistent lighting conditions were maintained to optimize image quality. Finally, digital images were captured and stored in JPEG format.

*Image processing with WinRHIZO and RootNav* Upon obtaining the digital images, they were systematically renamed according to their assigned identifiers. Using Adobe Photoshop CC 2019 software (Adobe, San Jose, CA, USA), tagged sections were cropped. The size of each cropped image was computed based on the markers. A deep learning-based root image segmentation tool developed in our laboratory (DeepLab V3C) was applied for root segmentation from the images [46]. RSA was extracted using WinRHIZO (Regent Instruments, Inc., Quebec City, Canada) [44, 47] and RootNav software [48–50]. Specific RSA values can be found in Supplementary Table 9.

Following imaging, fresh weights of above-ground and root components were obtained. Both above-ground and root were desiccated at 105 °C for 30 min, followed by drying to a constant weight at 80 °C to determine above-ground and root dry weights. Formulas for calculating the fresh root/shoot ratio, dry root/shoot ratio, water loss rate of the shoot, and water loss rate of the root are available in Supplementary Table 9.

### Yield, yield components and quality

Cotton bolls from 20 plants at the center of each plot were harvested for yield measurements. The bolls were harvested on October 18, 2021, and October 15, 2022. The harvested seed cotton was collected in nylon mesh bags and stored in a drying room for 20 days before weighing to determine the seed cotton yield. For cotton with a moisture content of less than 12%, weighing was carried out on the ginned cotton to determine the lint cotton yield.

### Statistical analysis

#### Drought tolerance evaluation

Drought tolerance coefficient (DTC), membership function, drought tolerance comprehensive evaluation values and other indicators were calculated as follows:

The drought resistance coefficient was the relative value of treatment and control, and the formula was as follows:

1 $$\mathrm{Drought}\;\mathrm{tolerance}\;\mathrm{coefficient}\;\left(\%\right)=\frac{X_i(\text{k})}{{CX}_i(\text{k})}$$

2 $$\mathrm{Coefficient}\;\mathrm{of}\;\mathrm{variation}\;\left(\%\right)=\frac{\mathrm{SD}}{\overline X}$$

Principal component analysis was performed on Drought tolerance coefficient of all traits, and then its membership function value $\mu \left({x}_{j}\right)$ was calculated:

3 $$\mu\left(x_{j}\right)=\frac{\left(x_{j}-x_{min}\right)}{\left(x_{max}-x_{min}\right)}\,\,\,\,\,j=1,2,\dots,n$$

The weight of each comprehensive index ${w}_{j}$ is calculated by the following formula:

4 $$\begin{array}{lc}w_j=p_j\sum\nolimits_{j=1}^np_j&j=1,2,\cdots,n\end{array}$$

D-value is the evaluation value of comprehensive drought resistance, the higher the Dj is, the material is indicated the greater the comprehensive drought resistance of the material. The calculation formula of D-value is as follows:

5 $$\begin{array}{lc}D=\sum\nolimits_{j=1}^n\lbrack u(x_j)\times w_j\rbrack&j=1,2,\cdots,n\end{array}$$

The formula for calculating the drought tolerance index (DTI) was as follows:

6 $$DTI=\frac{Value\;of\;under\;DS}{Value\;of\;under\;WW}\times\frac{Value\;of\;under\;DS}{Average\;value\;of\;all\;varieties\;unde\;rDS}$$

In order to comprehensively consider the root characteristics, the root traits were normalized and combined with the relative drought resistance coefficient of the traits to evaluate drought resistance.

7 $$Xi\mathrm{^{\prime}}\left(k\right)=\frac{\left[{X}_{i}\left(K\right)-{X}_{min}\right]}{\left[{X}_{max}-{X}_{min}\right]}$$

8 $$DTCi\mathrm{^{\prime}}=X{i}^{\mathrm{^{\prime}}}(k){ DTC}_{i}$$

Where, *Xi* (*k*) and CX*i* (*k*) represent the measured values of treatment and control traits, respectively; SD is the standard deviation; $\overline{X }$ is the average value of a trait; ${x}_{j}$ is the *i*th composite index; ${x}_{min}$ is the minimum value of the *i*th composite index;${x}_{max}$ is the maximum value of the *i*th composite index;${w}_{j}$ value represents the importance of the *i*th composite index in all composite indexes;${p}_{j}$ is the contribution rate of the ith comprehensive index of each variety. D-value is the comprehensive evaluation value of drought tolerance of all cultivars. According to the D-value of each variety, the drought tolerance of tested cotton cultivars can be classified. The DTI was employed to assess drought tolerance for achieving high cotton yields under drought stress. This index combines the drought tolerant coefficient under water stress. $X{i}^{\mathrm{^{\prime}}}(k)$ is the result of data normalization, and ${DTC}_{i}$ is the drought-resistance characteristic value of root system.

### Data analysis

Microsoft Excel 2019 (Statistical Product and Service Solutions) was used to record and sort general data. SPSS 26.0 (IBM Corp., Armonk, NY, USA) was used for ANOVA, PCA and data standardization. Microsoft Excel 2019 calculates membership functions and D values. Origin 2019b (OriginLab, Northampton, MA, USA) was used for graph plotting and hierarchical cluster analysis. Data are expressed as mean ± standard error (mean ± SE). Adobe Illustrator 2020 was used for representative image combinations. The D values were systematically clustered by using the Euclidean distance flat method and systematic clustering, and 80 cotton varieties could be clustered into 5 classes at a Euclidean distance of 0.2.

### Supplementary Information

**Additional file 1: Supplementary Fig. 1.** The soil relative water content in 2021 (A) and 2022 (B) in the experimental fields. WW, well-watered; DS, drought stress. **Supplementary Table 1.** Effects of drought stress on above-ground and root traits of cotton in 2021. **Supplementary Table 2.** Effects of drought stress on above-ground and root of cotton in 2022. **Supplementary Table 3.** Descriptive statistics of cotton yield (kg ha-1) under well-watered and drought stress conditions in 2021. **Supplementary Table 4.** Descriptive statistics of cotton yield (kg ha-1) under well-watered and drought stress conditions in 2022. **Supplementary Table 5.** Descriptive statistics of cotton aboveground boimass (g) under well-watered and drought stress conditions in 2021. **Supplementary Table 6.** Descriptive statistics of cotton aboveground boimass (g) under well-watered and drought stress conditions in 2022. **Supplementary Table 7.** The soil bulk density and field water capacity of the 0-20 cm, 20-40 cm and 40-60 cm. **Supplementary Table 8.** The names and authorized numbers of the different cotton cultivars. **Supplementary Table 9.** Root traits obtained from WinRHIZO and RootNav.

## Data Availability

Data will be made available on request. Contact details: Congcong Guo, 510807671@qq.com.

## Abbreviations

RSA: Root system architecture
PCA: Principal component analysis
D-value: Drought tolerance comprehensive evaluation values
Cl: Composite Index
WW: Well-watered
DS: Drought stress
DTI: Drought tolerance index
