# Supplementary material for: Evaluation of drought-tolerant varieties based on root system architecture in cotton (Gossypium hirsutum L.)
Source: BMC Plant Biol. 2024 Feb 21;24:127. doi: 10.1186/s12870-024-04799-x (PMC11295384; doi:10.1186/s12870-024-04799-x)
Supplement: Supplementary file 1 — Additional file 1: Supplementary Fig. 1. The soil relative water content in 2021 (A) and 2022 (B) in the experimental fields. WW, well-watered; DS, drought stress. Supplementary Table 1. Effects of drought stress on above-ground and root traits of cotton in 2021. Supplementary Table 2. Effects of drought stress on above-ground and root of cotton in 2022. Supplementary Table 3. Descriptive statistics of cotton yield (kg ha-1) under well-watered and drought stress conditions in 2021. Supplementary Table 4. Descriptive statistics of cotton yield (kg ha-1) under well-watered and drought stress conditions in 2022. Supplementary Table 5. Descriptive statistics of cotton aboveground boimass (g) under well-watered and drought stress conditions in 2021. Supplementary Table 6. Descriptive statistics of cotton aboveground boimass (g) under well-watered and drought stress conditions in 2022. Supplementary Table 7. The soil bulk density and field water capacity of the 0-20 cm, 20-40 cm and 40-60 cm. Supplementary Table 8. The names and authorized numbers of the different cotton cultivars. Supplementary Table 9. Root traits obtained from WinRHIZO and RootNav. [file 12870_2024_4799_MOESM1_ESM.zip › Supplementary figure legend+Supplementary tables.docx]

**Supplementary figure legend**

**Supplementary Fig. 1.** The soil relative water content of 2021 (A) and 2022 (B) in fields. WW, well-watered; DS, drought stress.

**Supplementary Table 1** Effects of drought stress on above-ground and root traits of cotton in 2021.

| Index^1)^ | Range | | Mean | | CV (%) | | DTC |
| --- | --- | --- | --- | --- | --- | --- | --- |
|  | WW | DS | WW | DS | WW | DS |  |
| Leaf area | 301.45-1991.98 | 18.10-886.59 | 895.82±125.30^a^ | 409.74±56.59^b^ | 34.96 | 13.25 | 0.48 |
| Plant height | 12.33-54.83 | 12.17-41.67 | 37.68±0.90 ^a^ | 24.33±0.91 ^b^ | 2.66 | 3.88 | 0.67 |
| SPAD | 38.63-51.63 | 24.33-47.00 | 46.09±1.47 ^a^ | 36.30±1.51 ^b^ | 3.17 | 4.30 | 0.79 |
| Canopy temperature | 27.43-34.83 | 29.87-39.10 | 30.03±0.45 ^b^ | 32.67±0.72 ^a^ | 1.47 | 2.17 | 1.09 |
| Leaf water potential | 3.33-5.35 | 6.31-9.02 | 4.34±0.63 ^b^ | 7.47±0.91 ^a^ | 14.65 | 12.24 | 1.74 |
| Relative water content | 0.74-0.96 | 0.41-0.92 | 0.84±0.08 ^a^ | 0.71±0.12 ^b^ | 9.06 | 17.58 | 0.81 |
| Root dry weight | 0.80-8.34 | 0.35-3.83 | 4.06±1.39 ^a^ | 2.16±0.63 ^b^ | 37.19 | 31.12 | 0.62 |
| Fresh root/shoot ratio | 0.04-0.86 | 0.03-0.71 | 0.19±0.10 ^b^ | 0.22±0.11 ^a^ | 50.07 | 45.39 | 1.41 |
| Dry root/shoot ratio | 0.09-0.38 | 0.07-0.25 | 0.13±0.02 | 0.15±0.04 | 17.34 | 23.84 | 1.21 |
| Water loss rate of shoot | 0.17-0.92 | 0.23-1.36 | 0.73±0.11 | 0.75±0.11 | 17.69 | 16.81 | 1.12 |
| Water loss rate of root | 0.03-0.94 | 0.13-1.51 | 0.68±0.14 | 0.71±0.12 | 23.79 | 18.63 | 1.46 |
| Total root length | 15.07-700.53 | 14.06-666.89 | 212.25±107.48 ^a^ | 119.24-52.02 ^b^ | 51.16 | 45.90 | 1.18 |
| Projection area | 5.11-547.93 | 4.26-207.41 | 75.47±54.38 ^a^ | 35.53±17.52 ^b^ | 57.74 | 47.85 | 1.17 |
| Surface area | 7.86-274.14 | 4.07-128.13 | 69.19±45.07 ^a^ | 34.32±16.68 ^b^ | 55.91 | 46.99 | 0.87 |
| Average diameter | 0.31-0.78 | 0.15-0.63 | 0.46±0.06 ^a^ | 0.40±0.06 ^b^ | 12.52 | 15.20 | 0.89 |
| Average volume | 0.60-34.49 | 0.30-9.01 | 5.53±3.94 ^a^ | 2.35±1.10 ^b^ | 57.40 | 44.77 | 0.76 |
| Average lateral root emergence angle | 51.39-84.66 | 47.46-76.17 | 67.35±5.95 ^a^ | 64.03±6.66 ^b^ | 9.04 | 10.52 | 0.96 |
| Average lateral root tip angle | 32.32-74.83 | 22.68-73.22 | 54.74±7.53 ^a^ | 48.78±8.68 ^b^ | 14.31 | 18.42 | 0.92 |
| Average length - all roots | 3.23-12.85 | 3.69-12.33 | 7.47±1.50 | 7.60±1.99 | 19.25 | 27.54 | 1.14 |
| Average length - all lateral roots | 1.71-11.37 | 2.24-11.18 | 6.15±1.57 | 6.43±1.79 | 26.85 | 28.90 | 1.29 |
| Lateral root count | 9-20 | 5-18 | 14±3 ^a^ | 11±3 ^b^ | 17.89 | 24.17 | 0.77 |
| Maximum width | 3.44-26.74 | 4.84-25.22 | 14.57±3.47 ^a^ | 11.61±3.22 ^b^ | 25.96 | 31.75 | 0.88 |
| Maximum depth | 7.32-30.01 | 11.35-29.74 | 22.84±3.69 ^a^ | 20.77±3.57 ^b^ | 18.11 | 18.48 | 0.94 |
| Width/depth ratio | 0.18-1.34 | 0.18-1.51 | 0.65±0.18 | 0.60±0.20 | 28.93 | 34.20 | 1.03 |
| Specific root length | 14.21-301.74 | 16.03-415.54 | 57.71±32.12 | 60.55±28.64 | 47.83 | 37.42 | 1.37 |
| Specific root surface area | 4.35-94.22 | 5.79-56.21 | 17.67±10.57 | 16.39±7.29 | 45.18 | 37.70 | 1.15 |
| Specific root volume | 0.24-5.75 | 0.21-3.02 | 1.28±0.75 | 1.10±0.47 | 45.76 | 38.79 | 1.05 |
| Root tissue density | 0.32-5.22 | 0.07-3.82 | 1.47±0.65 | 1.30±0.49 | 41.89 | 39.93 | 0.97 |

^1)^ Range, range of amplitude; Mean, mean value; CV, coefficient of variation; WW, well-watered; DS, drought stress; DTC, drought tolerant coefficient.

**Supplementary Table 2** Effects of drought stress on above-ground and root of cotton in 2022.

| Index^1)^ | Range | | Mean | | CV (%) | | DTC |
| --- | --- | --- | --- | --- | --- | --- | --- |
|  | WW | DS | WW | DS | WW | DS |  |
| Leaf area | 1093.08-5496.96 | 508.34-2379.74 | 2461.86±515.54^a^ | 1206.49±286.19^b^ | 20.81 | 23.67 | 0.54 |
| Plant height | 32.00-68.50 | 23.67-56.90 | 53.42±2.22 ^a^ | 42.22±2.20 ^b^ | 4.27 | 5.44 | 0.80 |
| SPAD | 47.43-56.70 | 36.03-54.10 | 52.22±1.13 ^a^ | 49.07±1.38 ^b^ | 2.17 | 2.88 | 0.94 |
| Canopy temperature | 25.00-29.13 | 26.50-30.50 | 27.78±0.20 ^b^ | 29.04±0.20 ^a^ | 0.75 | 0.70 | 1.05 |
| Leaf water potential | 3.44-6.43 | 6.24-9.39 | 4.93±0.36 ^b^ | 7.79±0.54 ^a^ | 7.32 | 7.06 | 1.62 |
| Relative water content | 0.79-1.29 | 0.54-0.92 | 0.88±0.06 ^a^ | 0.76±0.05 ^b^ | 6.06 | 6.94 | 0.87 |
| Root dry weight | 2.97-8.71 | 1.50-5.82 | 4.85±0.87 ^a^ | 3.18±0.69 ^b^ | 18.29 | 22.20 | 0.68 |
| Fresh root/shoot ratio | 0.05-0.42 | 0.06-0.76 | 0.16±0.04 ^b^ | 0.18±0.06 ^a^ | 21.93 | 31.13 | 1.19 |
| Dry root/shoot ratio | 0.04-0.49 | 0.06-0.58 | 0.16±0.05 ^b^ | 0.20±0.09 ^a^ | 27.70 | 39.14 | 1.29 |
| Water loss rate of shoot | 0.30-0.98 | 0.07-0.93 | 0.70±0.08 | 0.70±0.12 | 13.64 | 19.81 | 1.01 |
| Water loss rate of root | 0.60-0.92 | 0.59-0.86 | 0.73±0.07 | 0.73±0.07 | 9.58 | 10.08 | 1.00 |
| Total root length | 54.52-1238.44 | 94.51-1485.64 | 485.09±170.99 | 486.55±202.18 | 43.33 | 40.19 | 1.42 |
| Projection area | 63.23-319.58 | 70.55-263.32 | 178.83±29.95 ^a^ | 136.86±23.25 ^b^ | 16.87 | 17.15 | 0.80 |
| Surface area | 104.78-1431.75 | 68.45-1866.58 | 525.26±270.78 ^a^ | 489.82±248.35 ^b^ | 60.44 | 51.43 | 1.27 |
| Average diameter | 0.10-0.67 | 0.10-0.50 | 0.35±0.06 ^a^ | 0.32±0.04 ^b^ | 21.82 | 14.20 | 1.01 |
| Average volume | 7.25-157.21 | 6.72-352.78 | 53.20±23.22 ^a^ | 45.82±23.48 ^b^ | 54.74 | 49.31 | 1.23 |
| Average lateral root emergence angle | 54.36-78.93 | 36.22-79.24 | 68.22±5.62 ^a^ | 65.70±4.78 ^b^ | 8.35 | 7.66 | 0.97 |
| Average lateral root tip angle | 38.97-73.01 | 28.27-70.83 | 54.78±5.83 ^a^ | 49.95±6.01 ^b^ | 10.87 | 12.17 | 0.93 |
| Average length - all roots | 4.53-13.95 | 4.99-13.45 | 7.99±0.93 | 8.22±1.80 | 12.38 | 23.22 | 1.06 |
| Average length - all lateral roots | 3.67-12.99 | 5.18-14.27 | 7.33±0.96 ^b^ | 8.05±1.88 ^a^ | 14.12 | 23.98 | 1.13 |
| Lateral root count | 10-39 | 7-42 | 20±3 ^a^ | 17±3 ^b^ | 15.55 | 18.16 | 0.89 |
| Maximum width | 6.96-29.73 | 6.68-24.64 | 17.56±3.25 ^a^ | 13.92±2.93 ^b^ | 20.89 | 22.19 | 0.84 |
| Maximum depth | 14.66-27.70 | 9.67-25.53 | 21.77±2.03 ^a^ | 19.78±1.88 ^b^ | 9.86 | 9.72 | 0.92 |
| Width/depth ratio | 0.35-2.03 | 0.26-1.77 | 0.83±0.19 | 0.75±0.18 | 22.94 | 26.18 | 1.02 |
| Specific root length | 30.23-211.76 | 38.55-356.32 | 105.25±30.78 ^b^ | 148.91±51.19 ^a^ | 32.83 | 34.54 | 1.66 |
| Specific root surface area | 22.55-385.48 | 22.08-530.52 | 113.46±50.00 ^b^ | 159.90±75.84 ^a^ | 51.21 | 49.23 | 1.66 |
| Specific root volume | 2.34-25.89 | 3.08-55.24 | 11.41±4.28 ^b^ | 13.62±6.43 ^a^ | 43.31 | 45.28 | 1.30 |
| Root tissue density | 0.03-0.29 | 0.03-0.67 | 0.11±0.03 | 0.13±0.06 | 26.48 | 40.70 | 1.27 |

^1)^ Range, range of amplitude; Mean, mean value; CV, coefficient of variation; WW, well-watered; DS, drought stress; DTC, drought tolerant coefficient.

**Supplementary Table 3** Descriptive statistics of cotton yield (kg ha^-1^) under well-watered and drought stress conditions in 2021.

| Variety | WW | DS | Variety | WW | DS | Variety | WW | DS |
| --- | --- | --- | --- | --- | --- | --- | --- | --- |
| Jifeng 554 | 2925.0 ^a^ | 1282.5 ^b^ | Zhongmiansuo 60 | 2844.0 ^a^ | 1707.8 ^b^ | Jifeng1187 | 2874.9 ^a^ | 1050.0 ^b^ |
| Jifeng 103 | 3382.5 ^a^ | 2031.8 ^b^ | Xinshi 71143 | 3607.5 ^a^ | 2782.8 ^b^ | Jifeng 1458 | 2950.6 ^a^ | 2491.9 ^b^ |
| Jifeng 522 | 3600.0 ^a^ | 2034.0 ^b^ | Xinza 15 | 2917.5 ^a^ | 1866.0 ^b^ | Jifeng 103 | 4140.0 ^a^ | 1191.0 ^b^ |
| Jifeng 908 | 3826.8 ^a^ | 2536.5 ^b^ | Xinshi 17 | 2452.5 ^a^ | 1525.5 ^b^ | Jifeng 914 | 4140.0 ^a^ | 2639.3 ^b^ |
| Jifeng 914 | 3802.5 ^a^ | 2524.1 ^b^ | GK39 | 2371.0 ^a^ | 1238.4 ^b^ | Jifeng 965 | 4339.3 ^a^ | 2671.9 ^b^ |
| Jifeng 1982 | 3630.0 ^a^ | 2611.9 | 0 shi | 3142.5 ^a^ | 1028.6 ^b^ | MH335223 | 3870.0 ^a^ | 1625.6 ^b^ |
| Jifeng 4 | 3053.3 ^a^ | 2342.5 ^b^ | Zhongmiansuo 94A915 | 4731.4 ^a^ | 2898.0 ^b^ | Guoxinmian 11 | 2895.8 ^a^ | 1771.4 ^b^ |
| 7886 | 3528.0 ^a^ | 2032.5 ^b^ | Lumianyan 36 | 2745.0 ^a^ | 1027.5 ^b^ | Zhongmiansuo 17 | 3722.1 ^a^ | 2245.5 ^b^ |
| Cangmian 268 | 2220.0 ^a^ | 1265.6 ^b^ | DP33B | 3879.0 ^a^ | 1521.0 ^b^ | Chunbeibao | 3060.0 ^a^ | 999.0 ^b^ |
| Jimian 315 | 4042.5 ^a^ | 2320.7 ^b^ | Guoxinmian01 | 2497.5 ^a^ | 1980.0 ^b^ | Zhongmiansuo 60 | 3600.0 ^a^ | 2790.0 ^b^ |
| Han 218 | 3585.9 ^a^ | 1832.1 ^b^ | Guoxinmian02 | 3915.0 ^a^ | 3232.5 ^b^ | CG3020-3 | 3898.1 ^a^ | 1870.9 ^b^ |
| Hannong 12 | 3292.7 ^a^ | 1470.6 ^b^ | Guoxinmian03 | 3825.0 ^a^ | 2544.0 ^b^ | Jimian 2016 | 2421.0 ^a^ | 1390.5 ^b^ |
| Han 8266 | 3847.5 ^a^ | 1915.2 ^b^ | Guoxinmian05 | 3168.0 ^a^ | 1983.4 ^b^ | Ji 1518 | 3836.3 ^a^ | 1487.3 ^b^ |
| Han 258 | 2396.3 ^a^ | 1812.5 ^b^ | Hanwu 216 | 3120.0 ^a^ | 960.0 ^b^ | Jimian 262 | 3690.0 ^a^ | 1260.0 ^b^ |
| Han 686 | 3792.3 ^a^ | 2553.4 ^b^ | Zhongmian 100 | 2952.0 ^a^ | 1040.4 ^b^ | Ji 178 | 2988.8 ^a^ | 1472.1 ^b^ |
| YM111 | 2880.0 ^a^ | 2062.8 ^b^ | Zhongmiansuo 79 | 2829.4 ^a^ | 1957.9 ^b^ | Ji 172 | 3258.0 ^a^ | 3015.0 ^b^ |
| Nongda KZ05 | 4167.0 ^a^ | 1464.4 ^b^ | Cangmian 666 | 3259.5 ^a^ | 1620.9 ^b^ | Yuzaomian 9110 | 3222.0 ^a^ | 1811.3 ^b^ |
| Nongdamian 10 | 3420.0 ^a^ | 2238.8 ^b^ | Han 6203 | 3744.0 ^a^ | 3082.5 ^b^ | Dexiamian 1 | 4151.3 ^a^ | 1440.0 ^b^ |
| Nongdamian 12 | 4014.0 ^a^ | 2040.0 ^b^ | Shikang 126 | 3375.0 ^a^ | 1905.8 ^b^ | Jicai 6913 | 4260.6 ^a^ | 1665.0 ^b^ |
| Lumianyan 28 | 3175.7 ^a^ | 2290.0 ^b^ | Cang 198 | 2964.0 ^a^ | 395.1 ^b^ | Zhongmiansuo 23 | 3253.5 ^a^ | 849.4 ^b^ |
| Xuzhou 1818 | 3760.7 ^a^ | 3060.0 ^b^ | Ji 228 | 2925.0 ^a^ | 622.4 ^b^ | Zhongmiansuo 50 | 2814.8 ^a^ | 1485.0 ^b^ |
| Zhongmiansuo 41 | 3540.3 ^a^ | 1504.8 ^b^ | Guoxinmian 9 | 4099.5 ^a^ | 1504.8 ^b^ | Ji668 | 4160.7 ^a^ | 3288.0 ^b^ |
| Shandongxiamian11-42 | 2857.0 ^a^ | 1539.8 ^b^ | K836 | 4299.4 ^a^ | 1809.0 ^b^ | Zhibao 86-1 | 4192.5 ^a^ | 3352.5 ^b^ |
| Zhongmiansuo 12 | 3555.0 ^a^ | 2795.4 ^b^ | Lumian 522 | 2884.1 ^a^ | 1638.0 ^b^ | Jimian 958 | 4419.0 ^a^ | 1783.1 ^b^ |
| Yumian 19 | 3324.0 ^a^ | 2230.9 ^b^ | Lumian 5172 | 3926.3 ^a^ | 2025.0 ^b^ | Jifeng 1271 | 3906.0 ^a^ | 2561.0 ^b^ |
| Ejing 1 | 4053.4 ^a^ | 2144.6 ^b^ | K638 | 3334.5 ^a^ | 1125.0 ^b^ |  |  |  |
| Zhongmiansuo 35 | 2577.4 ^a^ | 1530.0 ^b^ | Guoxin 4 | 4003.5 ^a^ | 2610.0 ^b^ |  |  |  |

^a^ WW, well-watered; DS, drought stress.

**Supplementary Table 4** Descriptive statistics of cotton yield (kg ha^-1^) under well-watered and drought stress conditions in 2022.

| Variety | WW | DS | Variety | WW | DS | Variety | WW | DS |
| --- | --- | --- | --- | --- | --- | --- | --- | --- |
| Jifeng 554 | 3154.5 ^a^ | 1626.0 ^b^ | Zhongmiansuo 60 | 3526.5 ^a^ | 2098.1 ^b^ | Jifeng1187 | 3826.5 ^a^ | 1313.0 ^b^ |
| Jifeng 103 | 2830.0 ^a^ | 1710.0 ^b^ | Xinshi 71143 | 4074.8 ^a^ | 2448.0 ^b^ | Jifeng 1458 | 4531.5 ^a^ | 2251.5 ^b^ |
| Jifeng 522 | 1699.5 ^a^ | 987.4 ^b^ | Xinza 15 | 4847.4 ^a^ | 3063.6 ^b^ | Jifeng 103 | 4961.3 ^a^ | 1392.4 ^b^ |
| Jifeng 908 | 4347.0 ^a^ | 2773.3 ^b^ | Xinshi 17 | 3872.3 ^a^ | 2580.0 ^b^ | Jifeng 914 | 4169.3 ^a^ | 2831.1 ^b^ |
| Jifeng 914 | 2706.0 ^a^ | 1749.0 ^b^ | GK39 | 3687.4 ^a^ | 1534.5 ^b^ | Jifeng 965 | 1932.8 ^a^ | 1265.3 ^b^ |
| Jifeng 1982 | 3477.0 ^a^ | 2529.0 ^b^ | 0 shi | 4092.4 | 1694.0 ^b^ | MH335223 | 3997.5 ^a^ | 2071.5 ^b^ |
| Jifeng 4 | 4775.4 ^a^ | 3316.5 ^b^ | Zhongmiansuo 94A915 | 5693.4 ^a^ | 2646.8 ^b^ | Guoxinmian 11 | 4255.5 ^a^ | 2047.5 ^b^ |
| 7886 | 3388.5 ^a^ | 2080.8 ^b^ | Lumianyan 36 | 3454.2 ^a^ | 1737.0 ^b^ | Zhongmiansuo 17 | 5760.0 ^a^ | 3502.5 ^b^ |
| Cangmian 268 | 4243.5 ^a^ | 2522.3 ^b^ | DP33B | 6172.7 ^a^ | 2957.1 ^b^ | Chunbeibao | 3960.0 ^a^ | 1779.4 ^b^ |
| Jimian 315 | 4395.6 ^a^ | 2691.0 ^b^ | Guoxinmian01 | 3511.1 ^a^ | 1979.0 ^b^ | Zhongmiansuo 60 | 4831.7 ^a^ | 2873.6 ^b^ |
| Han 218 | 5589.0 ^a^ | 3214.5 ^b^ | Guoxinmian02 | 5044.5 ^a^ | 3861.0 ^b^ | CG3020-3 | 7905.0 ^a^ | 2691.0 ^b^ |
| Hannong 12 | 2927.3 ^a^ | 1244.0 ^b^ | Guoxinmian03 | 3663.0 ^a^ | 2520.0 ^b^ | Jimian 2016 | 3253.5 ^a^ | 1998.9 ^b^ |
| Han 8266 | 6097.5 ^a^ | 3201.0 ^b^ | Guoxinmian05 | 4903.9 ^a^ | 2687.1 ^b^ | Ji 1518 | 3850.5 ^a^ | 1902.2 ^b^ |
| Han 258 | 3354.8 ^a^ | 2319.8 ^b^ | Hanwu 216 | 3633.4 ^a^ | 1357.1 ^b^ | Jimian 262 | 2907.0 ^a^ | 1166.3 ^b^ |
| Han 686 | 2761.5 ^a^ | 1894.5 ^b^ | Zhongmian 100 | 4514.1 ^a^ | 1512.0 ^b^ | Ji 178 | 3345.8 ^a^ | 1375.5 ^b^ |
| YM111 | 3418.5 ^a^ | 2419.7 ^b^ | Zhongmiansuo 79 | 7605.0 ^a^ | 5220.0 ^b^ | Ji 172 | 2775.6 ^a^ | 2475.0 ^b^ |
| Nongda KZ05 | 3474.0 ^a^ | 1178.1 ^b^ | Cangmian 666 | 5415.0 ^a^ | 2833.7 ^b^ | Yuzaomian 9110 | 2660.1 ^a^ | 1096.7 ^b^ |
| Nongdamian 10 | 2587.5 ^a^ | 1615.9 ^b^ | Han 6203 | 4171.5 ^a^ | 3273.8 ^b^ | Dexiamian 1 | 4563.0 ^a^ | 2066.3 ^b^ |
| Nongdamian 12 | 6048.0 ^a^ | 3096.0 ^b^ | Shikang 126 | 3663.0 ^a^ | 2065.1 ^b^ | Jicai 6913 | 4833.0 ^a^ | 2291.6 ^b^ |
| Lumianyan 28 | 4503.6 ^a^ | 3303.0 ^b^ | Cang 198 | 4074.0 ^a^ | 1045.8 ^b^ | Zhongmiansuo 23 | 2709.0 ^a^ | 835.4 ^b^ |
| Xuzhou 1818 | 4371.0 ^a^ | 3582.0 ^b^ | Ji 228 | 2068.7 ^a^ | 662.4 ^b^ | Zhongmiansuo 50 | 3481.2 ^a^ | 1921.5 ^b^ |
| Zhongmiansuo 41 | 3784.5 ^a^ | 1458.0 ^b^ | Guoxinmian 9 | 4489.5 ^a^ | 1999.8 ^b^ | Ji668 | 4306.5 ^a^ | 3576.9 ^b^ |
| Shandongxiamian11-42 | 3241.8 ^a^ | 1848.0 ^b^ | K836 | 4557.0 ^a^ | 2326.5 ^b^ | Zhibao 86-1 | 3682.5 ^a^ | 2919.0 ^b^ |
| Zhongmiansuo 12 | 4108.5 ^a^ | 2496.0 ^b^ | Lumian 522 | 3136.5 ^a^ | 1305.0 ^b^ | Jimian 958 | 3463.9 ^a^ | 1701.6 ^b^ |
| Yumian 19 | 4104.0 ^a^ | 2835.0 ^b^ | Lumian 5172 | 6276.0 ^a^ | 3394.8 ^b^ | Jifeng 1271 | 3615.4 ^a^ | 1823.4 ^b^ |
| Ejing 1 | 2337.8 ^a^ | 1327.5 ^b^ | K638 | 4327.2 ^a^ | 1676.8 ^b^ |  |  |  |
| Zhongmiansuo 35 | 7114.5 ^a^ | 4405.5 ^b^ | Guoxin 4 | 3456.0 ^a^ | 2202.8 ^b^ |  |  |  |

**Supplementary Table 5** Descriptive statistics of cotton aboveground boimass (g) under well-watered and drought stress conditions in 2021.

| Variety | WW | DS | Variety | WW | DS | Variety | WW | DS |
| --- | --- | --- | --- | --- | --- | --- | --- | --- |
| Jifeng 554 | 27.37 ^a^ | 9.98 ^b^ | Zhongmiansuo 60 | 23.19 ^a^ | 10.97 ^b^ | Jifeng1187 | 37.69 ^a^ | 11.54 ^b^ |
| Jifeng 103 | 23.36 ^a^ | 11.58 ^b^ | Xinshi 71143 | 25.64 ^a^ | 18.62 ^b^ | Jifeng 1458 | 23.93 | 21.38 |
| Jifeng 522 | 23.59 ^a^ | 10.51 ^b^ | Xinza 15 | 25.72 ^a^ | 14.23 ^b^ | Jifeng 103 | 28.08 ^a^ | 7.13 ^b^ |
| Jifeng 908 | 22.39 ^a^ | 12.95 ^b^ | Xinshi 17 | 23.59 ^a^ | 12.64 ^b^ | Jifeng 914 | 25.10 ^a^ | 13.54 ^b^ |
| Jifeng 914 | 34.00 ^a^ | 19.36 ^b^ | GK39 | 21.28 ^a^ | 8.80 ^b^ | Jifeng 965 | 22.57 ^a^ | 11.78 ^b^ |
| Jifeng 1982 | 25.24 ^a^ | 16.07 ^b^ | 0 shi | 36.33 ^a^ | 10.89 ^b^ | MH335223 | 32.81 ^a^ | 12.62 ^b^ |
| Jifeng 4 | 23.64 ^a^ | 16.29 ^b^ | Zhongmiansuo 94A915 | 25.96 ^a^ | 13.27 ^b^ | Guoxinmian 11 | 29.76 ^a^ | 14.36 ^b^ |
| 7886 | 28.32 ^a^ | 13.90 ^b^ | Lumianyan 36 | 35.32 ^a^ | 12.63 ^b^ | Zhongmiansuo 17 | 28.07 ^a^ | 13.51 ^b^ |
| Cangmian 268 | 26.59 ^a^ | 12.15 ^b^ | DP33B | 37.15 ^a^ | 12.29 ^b^ | Chunbeibao | 32.57 ^a^ | 9.47 ^b^ |
| Jimian 315 | 18.04 ^a^ | 8.43 ^b^ | Guoxinmian01 | 21.89 | 20.38 | Zhongmiansuo 60 | 26.81 | 22.83 |
| Han 218 | 20.58 ^a^ | 8.43 ^b^ | Guoxinmian02 | 28.10 | 25.04 | CG3020-3 | 31.57 ^a^ | 12.45 ^b^ |
| Hannong 12 | 43.15 ^a^ | 14.53 ^b^ | Guoxinmian03 | 16.67 | 10.48 | Jimian 2016 | 42.64 ^a^ | 20.05 ^b^ |
| Han 8266 | 29.65 ^a^ | 11.97 ^b^ | Guoxinmian05 | 35.63 ^a^ | 19.11 ^b^ | Ji 1518 | 34.42 ^a^ | 12.42 ^b^ |
| Han 258 | 19.07 ^a^ | 12.95 ^b^ | Hanwu 216 | 39.65 ^a^ | 6.48 ^b^ | Jimian 262 | 29.72 ^a^ | 5.22 ^b^ |
| Han 686 | 30.00 ^a^ | 13.32 ^b^ | Zhongmian 100 | 27.56 ^a^ | 8.35 ^b^ | Ji 178 | 25.19 ^a^ | 10.03 ^b^ |
| YM111 | 17.26 ^a^ | 11.56 ^b^ | Zhongmiansuo 79 | 15.70 | 10.51 | Ji 172 | 17.93 | 15.66 |
| Nongda KZ05 | 30.44 ^a^ | 7.94 ^b^ | Cangmian 666 | 16.70 ^a^ | 6.73 ^b^ | Yuzaomian 9110 | 24.88 ^a^ | 11.01 ^b^ |
| Nongdamian 10 | 26.77 ^a^ | 15.73 ^b^ | Han 6203 | 24.31 | 21.21 | Dexiamian 1 | 21.06 ^a^ | 5.39 ^b^ |
| Nongdamian 12 | 28.34 ^a^ | 11.56 ^b^ | Shikang 126 | 23.99 | 21.01 | Jicai 6913 | 16.18 ^a^ | 5.16 ^b^ |
| Lumianyan 28 | 17.90 ^a^ | 11.07 ^b^ | Cang 198 | 22.04 ^a^ | 6.12 ^b^ | Zhongmiansuo 23 | 34.42 ^a^ | 7.04 ^b^ |
| Xuzhou 1818 | 16.32 ^a^ | 10.99 ^b^ | Ji 228 | 38.66 ^a^ | 10.55 ^b^ | Zhongmiansuo 50 | 15.93 ^a^ | 6.73 ^b^ |
| Zhongmiansuo 41 | 38.07 ^a^ | 12.61 ^b^ | Guoxinmian 9 | 43.04 ^a^ | 14.78 ^b^ | Ji668 | 23.50 ^a^ | 9.69 ^b^ |
| Shandongxiamian11-42 | 45.61 ^a^ | 20.05 ^b^ | K836 | 16.80 ^a^ | 6.48 ^b^ | Zhibao 86-1 | 25.10 ^a^ | 11.60 ^b^ |
| Zhongmiansuo 12 | 24.63 ^a^ | 17.31 ^b^ | Lumian 522 | 36.14 ^a^ | 17.02 ^b^ | Jimian 958 | 29.25 ^a^ | 11.01 ^b^ |
| Yumian 19 | 12.70 ^a^ | 7.48 ^b^ | Lumian 5172 | 29.45 ^a^ | 12.10 ^b^ | Jifeng 1271 | 32.210 ^a^ | 18.73 ^b^ |
| Ejing 1 | 35.51 ^a^ | 15.15 ^b^ | K638 | 41.60 ^a^ | 12.56 ^b^ |  |  |  |
| Zhongmiansuo 35 | 41.30 ^a^ | 20.38 ^b^ | Guoxin 4 | 38.310 ^a^ | 21.44 ^b^ |  |  |  |

**Supplementary Table 6** Descriptive statistics of cotton aboveground boimass (g) under well-watered and drought stress conditions in 2022.

| Variety | WW | DS | Variety | WW | DS | Variety | WW | DS |
| --- | --- | --- | --- | --- | --- | --- | --- | --- |
| Jifeng 554 | 16.82 ^a^ | 9.46 ^b^ | Zhongmiansuo 60 | 22.99 ^a^ | 13.71 ^b^ | Jifeng1187 | 113.09 ^a^ | 41.90 ^b^ |
| Jifeng 103 | 26.14 ^a^ | 16.32 ^b^ | Xinshi 71143 | 34.6 | 20.91 | Jifeng 1458 | 25.95 | 13.27 |
| Jifeng 522 | 54.98 ^a^ | 33.8 ^b^ | Xinza 15 | 37.75 | 25.68 | Jifeng 103 | 62.61 ^a^ | 7.40 ^b^ |
| Jifeng 908 | 49.79 ^a^ | 34.59 ^b^ | Xinshi 17 | 38.64 | 27.28 | Jifeng 914 | 60.96 ^a^ | 43.59 ^b^ |
| Jifeng 914 | 31.3 ^a^ | 21.83 ^b^ | GK39 | 31.87 ^a^ | 15.20 ^b^ | Jifeng 965 | 28.43 | 19.98 |
| Jifeng 1982 | 27.76 | 21.39 | 0 shi | 35.91 ^a^ | 16.27 ^b^ | MH335223 | 40.60 ^a^ | 21.25 ^b^ |
| Jifeng 4 | 35.37 | 28.66 | Zhongmiansuo 94A915 | 21.16 ^a^ | 9.80 ^b^ | Guoxinmian 11 | 33.29 ^a^ | 16.30 |
| 7886 | 24.89 ^a^ | 15.93 ^b^ | Lumianyan 36 | 21.55 ^a^ | 11.03 ^b^ | Zhongmiansuo 17 | 45.41 | 27.49 |
| Cangmian 268 | 25.70 | 15.89 | DP33B | 35.07 ^a^ | 17.08 ^b^ | Chunbeibao | 84.37 ^a^ | 40.73 ^b^ |
| Jimian 315 | 33.59 ^a^ | 20.62 ^b^ | Guoxinmian01 | 63.28 ^a^ | 37.03 ^b^ | Zhongmiansuo 60 | 41.29 ^a^ | 25.56 ^b^ |
| Han 218 | 43.69 ^a^ | 26.82 ^b^ | Guoxinmian02 | 48.59 | 44.42 | CG3020-3 | 39.19 ^a^ | 15.48 ^b^ |
| Hannong 12 | 58.55 ^a^ | 28.00 ^b^ | Guoxinmian03 | 45.59 | 34.2 | Jimian 2016 | 40.91 | 27.17 |
| Han 8266 | 29.9 ^a^ | 15.98 ^b^ | Guoxinmian05 | 59.18 ^a^ | 35.09 ^b^ | Ji 1518 | 29.96 ^a^ | 15.10 ^b^ |
| Han 258 | 31.91 | 24.79 | Hanwu 216 | 23.23 ^a^ | 7.67 ^b^ | Jimian 262 | 37.37 ^a^ | 16.44 ^b^ |
| Han 686 | 33.69 | 24.41 | Zhongmian 100 | 43.20 ^a^ | 16.11 ^b^ | Ji 178 | 22.94 ^a^ | 10.12 ^b^ |
| YM111 | 33.28 | 25.21 | Zhongmiansuo 79 | 29.79 | 21.96 | Ji 172 | 35.81 ^a^ | 29.51 |
| Nongda KZ05 | 33.26 ^a^ | 12.75 ^b^ | Cangmian 666 | 29.79 ^a^ | 15.70 ^b^ | Yuzaomian 9110 | 37.14 ^a^ | 16.82 ^b^ |
| Nongdamian 10 | 51.22 ^a^ | 34.81 ^b^ | Han 6203 | 54.92 | 45.87 | Dexiamian 1 | 38.32 ^a^ | 18.62 ^b^ |
| Nongdamian 12 | 31.79 ^a^ | 17.48 ^b^ | Shikang 126 | 50.80 ^a^ | 29.73 ^b^ | Jicai 6913 | 28.86 ^a^ | 13.71 ^b^ |
| Lumianyan 28 | 25.10 | 19.46 | Cang 198 | 51.05 ^a^ | 13.88 ^b^ | Zhongmiansuo 23 | 35.25 ^a^ | 8.07 ^b^ |
| Xuzhou 1818 | 13.74 | 9.69 | Ji 228 | 47.50 ^a^ | 17.15 ^b^ | Zhongmiansuo 50 | 32.80 ^a^ | 19.48 |
| Zhongmiansuo 41 | 34.16 ^a^ | 14.09 ^b^ | Guoxinmian 9 | 38.01 ^a^ | 18.31 ^b^ | Ji668 | 39.43 ^a^ | 35.22 |
| Shandongxiamian11-42 | 33.65 | 19.36 | K836 | 26.00 ^a^ | 14.74 ^b^ | Zhibao 86-1 | 33.23 ^a^ | 27.61 |
| Zhongmiansuo 12 | 29.43 | 26.19 | Lumian 522 | 10.74 ^a^ | 4.88 ^b^ | Jimian 958 | 65.39 ^a^ | 32.30 ^b^ |
| Yumian 19 | 65.16 ^a^ | 46.52 ^b^ | Lumian 5172 | 34.27 ^a^ | 20.16 ^b^ | Jifeng 1271 | 43.60 ^a^ | 24.53 ^b^ |
| Ejing 1 | 30.91 ^a^ | 17.64 ^b^ | K638 | 33.05 ^a^ | 14.18 ^b^ |  |  |  |
| Zhongmiansuo 35 | 35.92 ^a^ | 24.00 ^b^ | Guoxin 4 | 29.83 | 20.31 |  |  |  |

**Supplementary Table 7** The soil bulk density and field water capacity of the 0-20 cm, 20-40 cm and 40-60 cm.

| Soli layer (cm) | Soil Bulk density (g cm^-3^) | Field capacity (%) |
| --- | --- | --- |
| 0-20 | 1.33 | 34.9 |
| 20-40 | 1.52 | 35.2 |
| 40-60 | 1.53 | 35.3 |

**Supplementary Table 8** The names and authorized numbers of the different cotton cultivars.

| No. | Variety | Authorized number | No. | Variety | Authorized number |
| --- | --- | --- | --- | --- | --- |
| 1 | Jifeng 554 | Jishenmian 2009003 | 41 | Hanwu 216 | Jishenmian 2014010 |
| 2 | Jifeng 103 | Jishenmian 20190008 | 42 | Zhongmian 100 | Guoshenmian 2016003 |
| 3 | Jifeng 522 | Jishenmian 20050552 | 43 | Zhongmiansuo 79 | Yushenmian 2010006 |
| 4 | Jifeng 908 |  | 44 | Cangmian 666 | Lushenmian 20160030 |
| 5 | Jifeng 914 | Guoshenmian 2015003 | 45 | Han 6203 | Guoshenmian 2015002 |
| 6 | Jifeng 1982 | Jishenmian 2014001 | 46 | Shikang 126 | Guoshenmian 2008002 |
| 7 | Jifeng 4 | Guoshenmian 20210020 | 47 | Cang 198 | Jishenmian 2007006 |
| 8 | 7886 | GSM08003 | 48 | Ji 228 | Guoshenmian 2008003 |
| 9 | Cangmian 268 | Lushenmian 20160030 | 49 | Guoxinmian 9 | Guoshenmian 2009004 |
| 10 | Jimian 315 | Jishenmian 20190010 | 50 | K836 | Lushenmian 2012018 |
| 11 | Han 218 | Jishenmian 2015003 | 51 | Lumian 522 | Lushenmian 20170041 |
| 12 | Hannong 12 |  | 52 | Lumian 5172 |  |
| 13 | Han 8266 | Guoshenmian 2014001 | 53 | K638 | Lushenmian 2010010 |
| 14 | Han 258 | Jishenmian 2015009 | 54 | Guoxin 4 | Jishenmian 2006008 |
| 15 | Han 686 | Yushenmian 2011010 | 55 | Jifeng1187 | Jishenmian 20200005 |
| 16 | YM111 | Guoshenmian 20170002 | 56 | Jifeng 1458 | Jishenmian 20200004 |
| 17 | Nongda KZ05 | Jishenmian 2013006 | 57 | Jifeng 103 | Guoshenmian 20190014 |
| 18 | Nongdamian 10 | Jishenmian 2015007 | 58 | Jifeng 914 | Guoshenmian 2015003 |
| 19 | Nongdamian 12 | Jishenmian 2014011 | 59 | Jifeng 965 |  |
| 20 | Lumianyan 28 | Guoshenmian 2006012 | 60 | MH335223 | Jishenmian 20190018 |
| 21 | Xuzhou 1818 | I3A01080 | 61 | Guoxinmian 11 | Guoshenmian 2009001 |
| 22 | Zhongmiansuo 41 | Guoshenmian 2002001 | 62 | Zhongmiansuo 17 | xinshemmian19980071991 |
| 23 | Shandongxiamian11-42 |  | 63 | Chunbeibao |  |
| 24 | Zhongmiansuo 12 | Lushenmian 0064 | 64 | Zhongmiansuo 60 | Zheshenmian 2007002 |
| 25 | Yumian 19 | Guoshenmian 2001002 | 65 | CG3020-3 |  |
| 26 | Ejing 1 | GS08002-1991 | 66 | Jimian 2016 | Jishenmian 20199002 |
| 27 | Zhongmiansuo 35 | Guoshenmian 990005 | 67 | Ji 1518 | Jishenmian 2014005 |
| 28 | Zhongmiansuo 60 | Shanshenmian 2013001 | 68 | Jihang 8 | Jishenmian 20190009 |
| 29 | Xinshi 71143 | Guoshenmian 2014004 | 69 | Jimian 262 | Jishenmian 20200002 |
| 30 | Xinza 15 | Jishenmian 2014007 | 70 | Ji 178 | Jishenmian 2015010 |
| 31 | Xinshi 17 | Jishenmian 2015004 | 71 | Ji 172 | Jishenmian 20210003 |
| 32 | GK39 | Guoshenmian 2015008 | 72 | Yuzaomian 9110 | Yushenmian 2012006 |
| 33 | 0 shi |  | 73 | Dexiamian 1 | Lushenmian 0220 |
| 34 | Zhongmiansuo 94A915 | Jinshenmian 2016002 | 74 | Jicai 6913 |  |
| 35 | Lumianyan 36 | Lushenmian 2009022 | 75 | Zhongmiansuo 23 | Guoshenmian 980007 |
| 36 | DP33B |  | 76 | Zhongmiansuo 50 | Guoshenmian 2007013 |
| 37 | Guoxinmian01 | Yushenmian 2009004 | 77 | Ji668 | Guoshenmian 2001001 |
| 38 | Guoxinmian02 |  | 78 | Zhibao 86-1 | GS08009-1984 |
| 39 | Guoxinmian03 | Guoshenmian 2006003 | 79 | Jimian 958 | Guoshenmian 2006005 |
| 40 | Guoxinmian05 | Guoshenmian 2006003 | 80 | Jifeng 1271 | Jishenmian 2012002 |

**Supplementary Table 9** Root traits obtained from WinRHIZO and RootNav.

| Trait | Data obtained by | Description |
| --- | --- | --- |
| Total root length | WinRHIZO REG2009 | Cumulative length of all the roots per plant (cm) |
| Projected area | WinRHIZO REG2009 | Projected area of all roots per plant (cm^2^) |
| Superficial area | WinRHIZO REG2009 | Superficial area of all roots per plant (cm^2^) |
| Average diameter | WinRHIZO REG2009 | Average diameter of all roots per plant (mm) |
| Average volume | WinRHIZO REG2009 | Average volume of all roots per plant (cm^3^) |
| Average lateral root emergence angle | RootNav | Root angles along all lateral root ranges (°) |
| Average lateral root tip angle | RootNav | Root angle of lateral apical root (°) |
| Average length-all roots | RootNav | The average length of all primary and lateral roots (cm) |
| Average length-lateral roots | RootNav | Mean length of all lateral roots in centimeters (cm) |
| Lateral root count | RootNav | The number of all lateral root (NO. plant) |
| Maximum width | RootNav | The maximum horizontal width of the whole RSA (cm) |
| Maximum depth | RootNav | The maximum vertical distance reached by the root system (cm) |
| Width/depth ratio | RootNav | The ratio of maximum width to deep (Ratio) |
| Fresh root/shoot ratio | Calculated | $\frac{Fresh weight under ground}{Fresh weight above ground}$ (%) |
| Dry root/shoot ratio | Calculated | $\frac{Under ground dry weight}{Above ground dry weight}$ (%) |
| Water loss rate of shoot | Calculated | $1 -\frac{Dry weight of above ground}{Fresh weight of above ground}$ |
| Water loss rate of root | Calculated | $1 -\frac{Dry weight of under ground}{Fresh weight of under ground}$ |
| Specific root surface area | Calculated | $\frac{Total root surface area}{Root dry weight}$ (cm g^-2^) |
| Specific root volume | Calculated | $\frac{Total root volume}{Root dry weight}$ (cm g ^-3^) |
| Specific root length | Calculated | $\frac{Total root length}{Root dry weight}$ (cm g^-1^) |
| Root tissue density | Calculated | $\frac{Dry root mass}{Total root volume}$ (g cm^-3^) |
