# Supplementary figures and images for: Evaluation of drought-tolerant varieties based on root system architecture in cotton (Gossypium hirsutum L.)
Source: BMC Plant Biol. 2024 Feb 21;24:127. doi: 10.1186/s12870-024-04799-x (PMC11295384; doi:10.1186/s12870-024-04799-x)

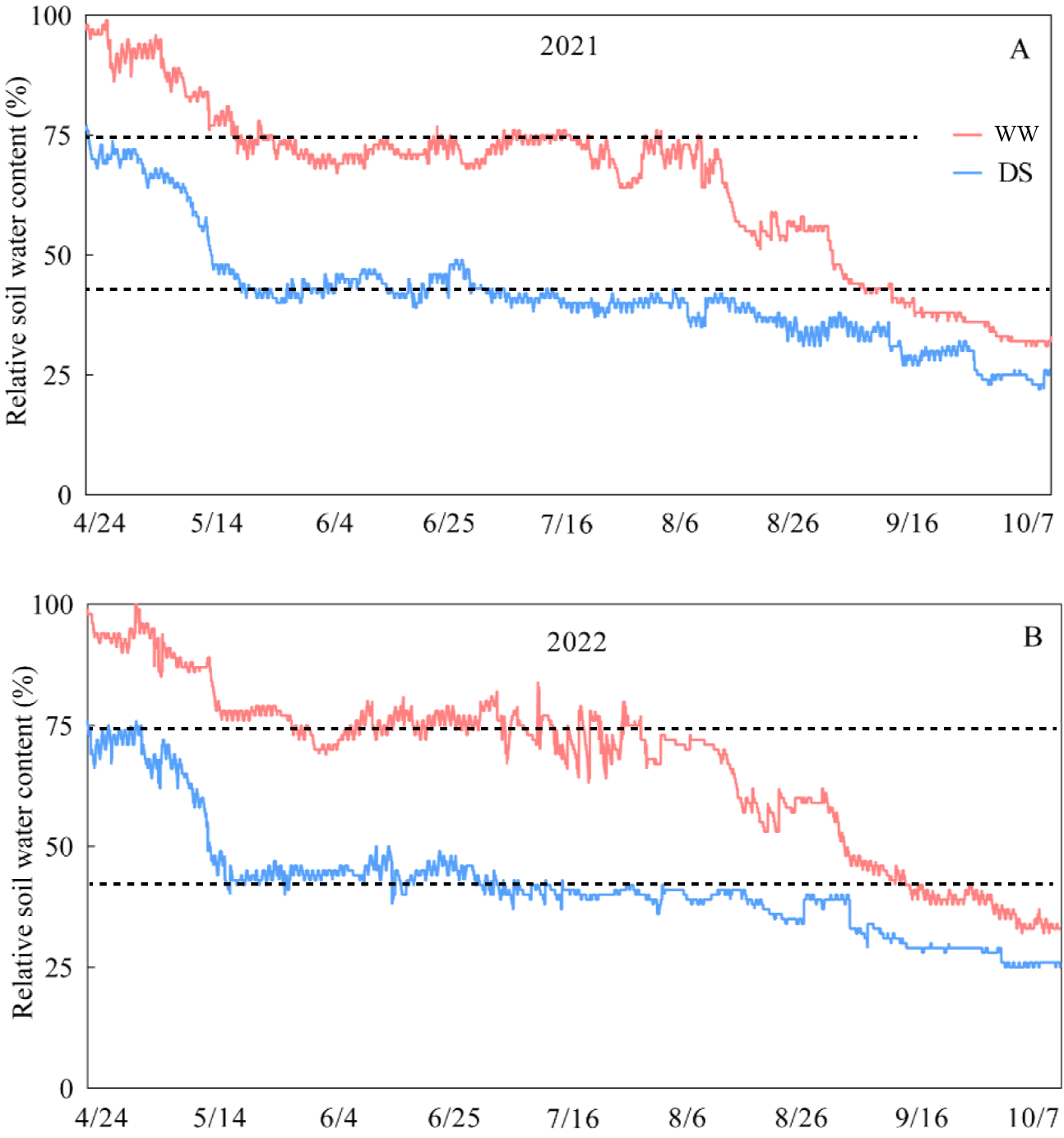

Supplement: Supplementary file 1 — Additional file 1: Supplementary Fig. 1. The soil relative water content in 2021 (A) and 2022 (B) in the experimental fields. WW, well-watered; DS, drought stress. Supplementary Table 1. Effects of drought stress on above-ground and root traits of cotton in 2021. Supplementary Table 2. Effects of drought stress on above-ground and root of cotton in 2022. Supplementary Table 3. Descriptive statistics of cotton yield (kg ha-1) under well-watered and drought stress conditions in 2021. Supplementary Table 4. Descriptive statistics of cotton yield (kg ha-1) under well-watered and drought stress conditions in 2022. Supplementary Table 5. Descriptive statistics of cotton aboveground boimass (g) under well-watered and drought stress conditions in 2021. Supplementary Table 6. Descriptive statistics of cotton aboveground boimass (g) under well-watered and drought stress conditions in 2022. Supplementary Table 7. The soil bulk density and field water capacity of the 0-20 cm, 20-40 cm and 40-60 cm. Supplementary Table 8. The names and authorized numbers of the different cotton cultivars. Supplementary Table 9. Root traits obtained from WinRHIZO and RootNav. [file 12870_2024_4799_MOESM1_ESM.zip › Supplementary Fig. 1. The soil relative water content.tif]
